# Robust and Interpretable Deep Learning Fault Diagnosis in Complex Chemical Processes: Performance Enhancement via Dempster–Shafer Theory and Feature Attention

**DOI:** 10.1021/acsomega.6c01554

**Published:** 2026-07-09

**Authors:** Remigius Nnadozie Ewuzie, Shivaneswar Gunasekaran, Zainal Ahmad, Juwari Juwari, Norazwan Md Nor

**Affiliations:** School of Chemical Engineering, Engineering Campus, Universiti Sains Malaysia, 14300 Nibong Tebal, Penang, Malaysia

## Abstract

Monitoring and diagnosing faults in chemical processes is essential for ensuring operational safety and product quality, particularly as industrial systems become increasingly complex. Deep learning has emerged as an effective data-driven approach for handling high-dimensional and nonlinear process data. Although effective at feature extraction, deep learning models face challenges related to sensor uncertainty, conflicting diagnostic signals, and limited interpretability. In response to these limitations, this study presents a novel fault diagnosis framework that integrates autoencoder (AE) and long short-term memory (LSTM) models with Dempster–Shafer theory (DST) and feature attention mechanisms. The framework combines deep learning’s representation learning capability with DST’s ability to manage uncertainty, while feature attention enhances interpretability. Validation was conducted using the Tennessee Eastman process (TEP) dataset, during which four key hyperparameters were carefully tuned. The standalone AE and LSTM models attained accuracies of 85 and 70%, respectively, which improved to 91 and 75% after DST integration. The AE–DST approach achieved a precision of 92%, outperforming conventional techniques. In addition, the attention-enhanced AE model achieved strong performance (accuracy: 90%, precision: 91%, F1-score: 90%), while feature attention identified critical process variables and improved diagnostic transparency.

## Graphical abstract

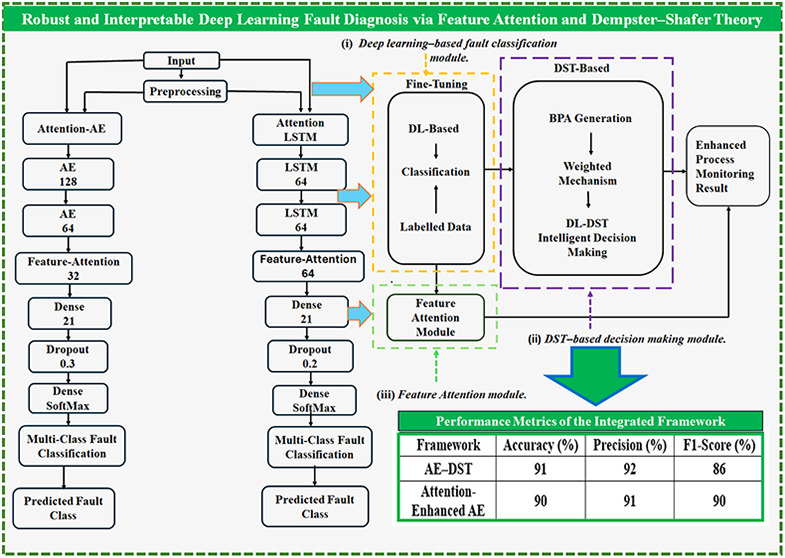

## 1 Introduction

Recent developments in modern industrial technologies have played a key role in advancing the automation of chemical processes.^1,2^ While automation improves stability, efficiency, and response speed, incidents involving equipment failures, environmental hazards, and human casualties still occur despite advanced control and safety systems,^3,4^ these issues stem from the reliance on human operators to detect and respond to abnormalities in complex, large-scale chemical systems, making process monitoring and fault diagnosis essential for safe, stable, and efficient operations.^5^

### 1.1 Background

Process monitoring tracks system variables for deviations, while fault diagnosis identifies fault types and causes as a multiclass problem. Together, they form an intelligent, real-time framework that enables early detection, timely corrective action, and minimal disruption while enhancing reliability, preventive maintenance, and overall performance.^6^ Monitoring and fault diagnosis methodologies are commonly categorized into three main approaches: model-based,^7^ knowledge-based,^8^ and data-driven.^9^ Unlike knowledge-based and model-based approaches that depend heavily on the detailed physicochemical understanding of the process, data-driven approaches construct fault diagnosis models by identifying significant patterns within historical process data.^7^ Given the complexity, nonlinearity, and dynamic nature of large-scale industrial systems, developing accurate models or relying solely on expert knowledge is often impractical.

The rapid advancements in industrial sensing and automation technologies have led to the continuous generation of vast amounts of real-time operational data in modern chemical plants.^10,11^ These rich datasets provide deep insights into system behavior and dynamics, making data-driven approaches increasingly attractive. Data-driven fault monitoring has gained attention for its scalability and effectiveness in complex industrial processes. Extracting relevant information from highly correlated, redundant sensor data is crucial for building robust, accurate, and reliable diagnostic models.^12^ In recent years, numerous feature extraction techniques have been introduced using ML and multivariate statistical approaches. Among the most commonly employed linear methods are canonical correlation analysis (CCA),^13^ principal component analysis (PCA),^14–17^ Fisher discriminant analysis (FDA),^18,19^ and partial least-squares (PLS).^20^

To capture more complex data patterns, nonlinear approaches such as kernel PCA,^19^ kernel PLS,^21^ artificial neural network (ANN),^22,23^ and support vector machine (SVM)^24,25^ have also been utilized. However, their ability to represent and generalize from large-scale, high-dimensional process data is limited, particularly in the Big Data era. In comparison, deep-learning models with multiple layers provide enhanced representational capabilities, allowing for the extraction of richer and more discriminative features from highly complex chemical processes. Deep learning models with multiple layers extract richer and more discriminative features from complex chemical processes. Early training challenges, like vanishing and exploding gradients, were overcome by Hinton et al.,^26^ using unsupervised layer-wise pretraining followed by backpropagation fine-tuning. Since then, deep learning^27,28^ has excelled in diverse fields by effectively capturing complex feature representations.

### 1.2 Limitations and Motivation

Despite significant advances in deep learning architectures, such as autoencoders (AEs), long short-term memory (LSTM) networks, and gated recurrent units (GRUs), for fault detection and diagnosis (FDD), their practical application in complex chemical process environments remains limited by several fundamental challenges. These challenges motivate the development of more robust, uncertainty-aware, and interpretable monitoring frameworks, as discussed in the following.

#### 1.2.1 Limited Development of Multiarchitecture Deep Learning Frameworks

Most existing FDD studies rely on a single deep-learning architecture, limiting their ability to capture complex spatiotemporal characteristics in chemical processes. As a result, these models often struggle to distinguish multiple fault types under nonlinear dynamics, strong variable coupling, overlapping fault signatures, and time-varying operating conditions, leading to degraded diagnostic performance and reduced robustness in multifault scenarios.

#### 1.2.2 Lack of Systematic Hyperparameter Tuning

The performance of DL models for fault detection and diagnosis is highly sensitive to architectural choices and hyperparameters, such as the learning rate, number of layers, and epochs. Many studies neglect systematic tuning, resulting in reduced performance and limited reproducibility. This limitation highlights the need for structured hyperparameter optimization to ensure consistent, high-accuracy fault classification in complex chemical processes.

#### 1.2.3 Limited Integration of Deep Learning with Uncertainty-Aware Evidence Fusion

Previous studies have explored the application of Dempster–Shafer theory (DST) for fault diagnosis. For instance, Wang et al.^29^ applied DST to fuse outputs from multiple classifiers to improve diagnostic accuracy, while Ghosh et al.^30^ utilized DST-based evidence fusion for fault identification using statistical features. In both cases, the fused evidence is derived from shallow model outputs or handcrafted features rather than deep-learned representations. However, existing approaches do not fully exploit deep learning’s ability to extract high-level latent features and capture complex nonlinear and temporal dependencies, and they often lack adaptive feature weighting prior to fusion. These limitations motivate an integrated framework in which models such as AE and LSTM generate more informative, structured, and adaptive evidence representations, further enhanced by feature attention mechanisms for adaptive weighting.

#### 1.2.4. Lack of Interpretability and Transparency in Diagnostic Decisions

Most deep learning–based FDD methods operate as black boxes, providing little insight into the variables or temporal patterns driving fault predictions. This limits operator trust and practical adoption. Integrating feature attention mechanisms can highlight fault-relevant variables and temporal states, enabling transparent, explainable, and reliable diagnostic decisions.

### 1.3 Contributions and Innovations

This study addresses the major limitations of existing deep learning FDD frameworks in complex chemical processes. The main contributions of this work are as follows:

1. Development of advanced deep learning architectures for multifault diagnosis.
  Formulation and implementation of complementary deep learning architectures capable of capturing complex spatiotemporal dependencies to accurately identify and classify multiple fault types in highly nonlinear chemical-process environments.
2. Systematic hyperparameter optimization was used for improved model performance.
  Enhancement of model accuracy and reproducibility through structured hyperparameter tuning ensures robust and consistent fault classification across varying process conditions.
3. Fault confidence evaluation:
  Implementation of fault confidence assessment using basic probability assignment (BPA) and accuracy-based weighting to enhance reliability in multisensor fault diagnosis.
4. Integration of deep learning with Dempster–Shafer theory.
  A novel framework is developed in which AE and LSTM models generate high-level latent and temporal representations as evidence of DST-based fusion. Unlike conventional approaches that rely on classifier outputs or handcrafted features, the proposed method utilizes deep learned representations with feature attention to produce adaptively weighted evidence.
5. Enhanced interpretability was achieved through feature attention mechanisms.
  Integration of feature attention highlights fault-relevant process variables and temporal states, providing transparent, explainable, and trustworthy diagnostic decisions.

These innovations establish an uncertainty-aware and interpretable FDD framework that addresses key challenges in multifault diagnosis. First, an attention mechanism is incorporated to achieve variable-level interpretability, enabling the identification of the most influential process variables contributing to fault decisions. Second, the framework establishes a strong integration between deep feature learning and Dempster–Shafer theory, where learned feature representations are directly transformed into basic probability assignments for uncertainty-aware fusion, rather than relying on decision-level fusion. These features distinguish the proposed framework from conventional DST-based classifier fusion and hierarchical distributed diagnosis methods, which typically lack both deep representation–evidence integration and inherent interpretability. Furthermore, the framework advances evidence modeling from shallow outputs to deep, structured representations.

### 1.4 Organization

The remainder of this article is organized as follows: Section 2 discusses related studies. Section 3 presents a detailed explanation of the methodology, including the attention mechanism, deep learning models, and Dempster–Shafer theory employed. In Section 4, the proposed framework, integrating deep learning with DST and feature attention, is experimentally validated using the Tennessee Eastman process (TEP) benchmark. Finally, Section 5 summarizes the key contributions of the study and outlines potential directions for future research.

## 2 Related Works

Recently, DL models have been applied in chemical industries for process modeling and fault diagnosis.^31,32^ Jiang et al.^33^ applied a stacked denoising autoencoder (SDAE) to construct a fault diagnosis model, incorporating an active learning mechanism that enables the model to selectively acquire relevant data for online refinement. Similarly, Zhang and Zhao^34^ introduced a deep belief network (DBN) designed to classify faults in the well-known Tennessee Eastman process, utilizing mutual information for feature extraction and developing DBN submodels for each operational state. Moreover, Jiang et al.^35^ also implemented stacked sparse autoencoders (SSAE) for fault identification in the TE process, taking into account its dynamic characteristics and proposing a semisupervised learning strategy. Wu and Zhao^1^ further explored the use of convolutional neural networks (CNNs) for fault classification in the TE process.

Arunthavanathan and his group^36^ developed a CNN–LSTM model for early fault detection in multivariate systems, effectively predicting process parameters and identifying faults in the Tennessee Eastman process. The stacked autoencoder is also widely used for unsupervised process monitoring. In related work, Tao et al.^37^ proposed a stacked autoencoder–SoftMax framework and an ensemble of 10 deep networks to improve fault diagnosis and enhance generalization. Similarly, Hosseini-Asl et al.^38^ developed a novel autoencoder network trained under non-negativity constraints, enabling the model to extract meaningful features from partial or incomplete data representations. More recently, Ying et al.^39^ employed stacked supervised autoencoders (SSAE) to extract deep fault-relevant features by performing layer-wise pretraining followed by fine-tuning, thereby enhancing classification performance for fault detection in the TEP. Furthermore, Yu and Yan^40^ employed DBNs to enhance abnormal fluctuation patterns, thereby improving feature quality for fault detection.

DST handles uncertainty and fuses noisy sensor data, making it useful for multisensor fault diagnosis. For example, Basir and Yuan^41^ applied DST to integrate neural network outputs into BPAs, combining multiple local models to identify fault types and locations. Similarly, Tabassian et al.^42^ introduced a supervised learning approach within the DST framework to address uncertainty in sensor data by applying a relabeling strategy that refines labels based on proximity to predefined class prototypes. Jiang et al.^43^ proposed a DST-based method for weld defect classification that integrates the analytic hierarchy process (AHP) to assign feature weights to sensor inputs, enhancing classification accuracy. Jiang et al.^44^ proposed an evidence fusion framework that builds the frame of discernment from time-series sensor data to improve diagnostic precision, while Liu et al.^45^ used weighted sensor fusion with ER, optimizing weights to match training outcomes. Table 1 summarizes the advantages and limitations of process monitoring methods.

**Table 1:** Summary of Strengths and Limitations of Process Monitoring and Fault Diagnosis Methods

methods	strengths	limitations	references
distributed control systems	real-time monitoring and control, well-integrated in industrial plants.	limited fault diagnosis capability, difficult to adapt to novel fault scenarios.	^4^
model-based	rely on first principles, offer high interpretability and physical relevance.	require accurate system models, challenging for complex nonlinear systems.	^7^
knowledge-based	use expert rules and heuristics, interpretable and easy to implement.	dependent on expert knowledge, may not generalize to unseen faults.	^8^
multivariate statistical	handle correlated variables, useful for dimensionality reduction.	assume linearity, struggle with dynamic or nonlinear behavior.	^15^
machine learning	effective for classification and regression tasks, learn from historical data.	require feature engineering, may not capture complex temporal dynamics.	^26^
deep learning	automatically extract features, strong in handling high-dimensional time series.	struggle with uncertainty, sensor noise, and missing data.	^27^
evidential networks	combine multiple sources of information under uncertainty.	construction and computation of mass functions can be complex.	^46^
belief entropy	quantifies uncertainty in belief distributions, aids in decision refinement.	interpretation and integration into larger systems may be nontrivial.	^47^
Bayesian inference	naturally handles uncertainty and updates beliefs as new data becomes available.	computationally expensive for large-scale or high-dimensional problems.	^15^

## 3 Methodology

### 3.1 Attention Mechanism

The attention mechanism is a key component in modern deep learning, inspired by the human visual and cognitive systems’ ability to selectively focus on salient information while suppressing irrelevant stimuli. Subsequently introduced into machine learning, the attention mechanism has become a core technique in domains such as natural language processing and computer vision, owing to its effectiveness in enhancing feature representation and model interpretability.^48,49^ In general, attention operates by assigning adaptive importance weights to input features based on their relevance to the current task. The widely adopted scaled dot-product attention can be expressed as

1 $${attention}_{output}=\left(Q,K,V\right)=SoftM\mathrm{ax}\frac{QK^{T}}{\sqrt{d_{k}}})\times V$$

*Q*, *K*, and *V* represent query, key, and value matrices, and *d*_*k*_ defines the dimensionality of the associated key vector.

This formulation captures complex dependencies by computing query–key similarities, normalizing them via SoftMax, and applying attention weights to values. In fault diagnosis, attention adaptively emphasizes fault-relevant features, reducing noise and irrelevant variations. Integrated with models like autoencoders and LSTMs, it highlights critical temporal states, capturing long-range dependencies and context-dependent fault signatures for improved robustness and scalability (Figure 1).

**Figure 1:**
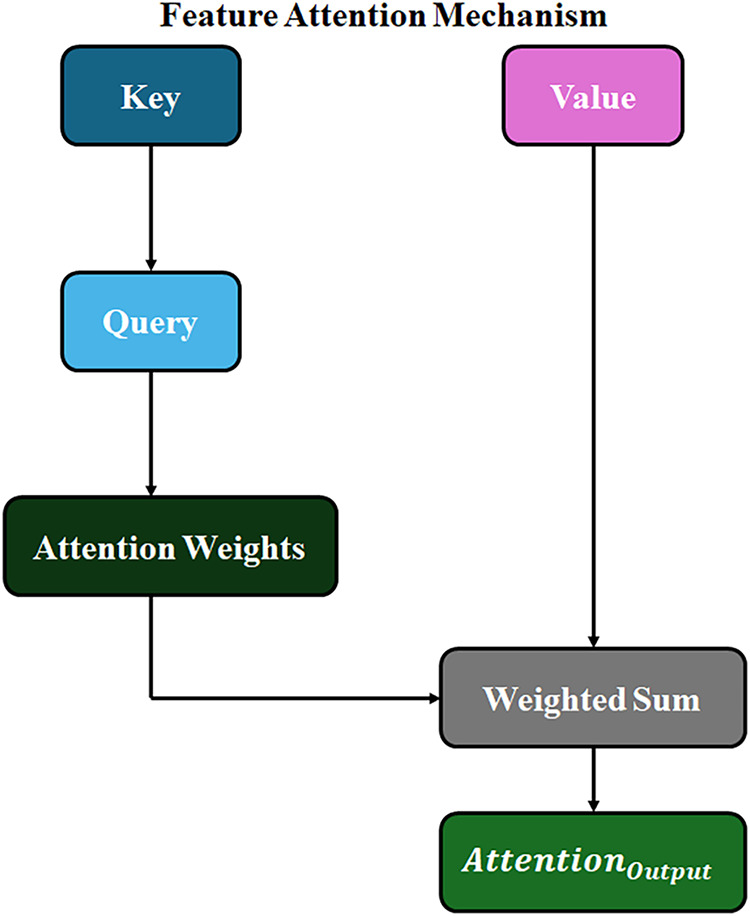
Illustration of the feature attention mechanism.

### 3.2 Deep Learning Models

In process industries, deep learning architectures such as autoencoders, CNNs, and LSTMs effectively capture nonlinear relationships, enabling accurate process prediction and early fault detection.^50^ DL models can be supervised, mapping inputs to known fault types for classification, or unsupervised, detecting anomalies in unlabeled data.^51^

#### 3.2.1 Autoencoder

An autoencoder (AE)^52^ is a widely utilized unsupervised ML model that employs neural networks to learn compact data representations. Structurally, it is composed of three layers: an input layer, a hidden (or encoding) layer, and an output (or decoding) layer, as illustrated in Figure 2. The goal of an autoencoder is to reconstruct the input at the output, meaning that the input and output layers have the same number of neurons. The hidden layer compresses the inputs into a lower-dimensional representation. Autoencoders, trained via backpropagation like feedforward networks, encode inputs into a feature space and decode them to reconstruct the original data. These processes are expressed mathematically as follows

2 $$y^{T}=f\left(\mathrm{wx}+b\right)$$

where *w* and *b* represent the learnable parameters, *f* denotes the activation function, *x* is the input vector, and *y* corresponds to the encoded (hidden) representation. During the decoding stage

3 $$x^{T}=f\left(w^{T}b^{T}+c\right)$$

where *w* ^*T*^ is the transpose of the weight matrix, *b* ^*T*^ is the bias associated with the output layer, *x* ^*T*^ is the reconstructed version of the input at the output layer, and *c* is an optional additional bias.

**Figure 2:**
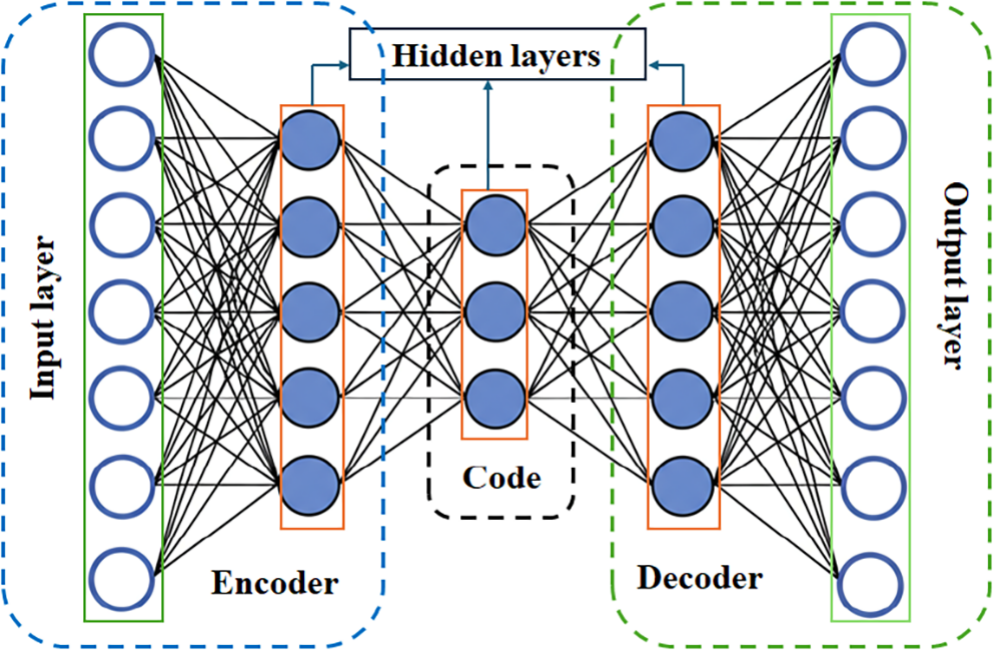
Structure of an autoencoder featuring three neurons in the hidden layer.

This equation computes the reconstructed output *x* ^*T*^ by linearly transforming the hidden representation using the transpose of the weight matrix and the output bias *b* ^*T*^ followed by the application of a nonlinear activation function. The goal is to reconstruct the input with minimal error, updating the autoencoder’s parameters as follows

4 $$w_{\mathrm{up}}=w-\left(\eta \frac{\partial E}{\partial w}\right)$$

5 $$b_{\mathrm{up}}=b-\left(\eta \frac{\partial E}{\partial b}\right)$$

where *w*_up_ and *b*_up_ are the updated values of the weight and bias parameters after one training step. *w* and *b* are the current weight and bias parameters. η represents the learning rate, a positive scalar governing the update step size. $\frac{\partial E}{\partial w}$ and $\frac{\partial E}{\partial b}$ represent the weight and bias gradients of the cost function *E*. The same weights are used for encoding and decoding, with training aiming to minimize the loss function.

6 $$J\left(w,b\right)=\frac{1}{N}\sum_{n=1}^{N}({x-y)}^{2}$$

where *N* refers to the total training samples, while *x* and *y* represent the input and output vectors, respectively. (*x*–*y*)^2^ is the squared error between the input and its reconstruction. $\frac{1}{N}$ is the average over the entire dataset. *J*(*w*, *b*) is the total loss function. Model parameters (*w*, *b*) are optimized via backpropagation to minimize loss, with the Adam algorithm, a gradient descent-based method, used for training.

#### 3.2.2 Long Short-Term Memory Network

The long short-term memory network^53^ overcomes the vanishing gradient problem in RNNs and effectively captures long-term temporal dependencies for fault diagnosis. Its memory cell and three nonlinear gates enable learning of both short and long-term patterns in multisensor data, as shown in Figure 3. The forget gate, the input gate, and the output gate. These gates are computed using the current input *x*_*t*_ and the previously hidden state *h*_(*t*–1)_, undergo a linear transformation, and are then processed by a sigmoid activation function, which scales the output to a range between 0 and 1 to regulate the flow of information.

**Figure 3:**
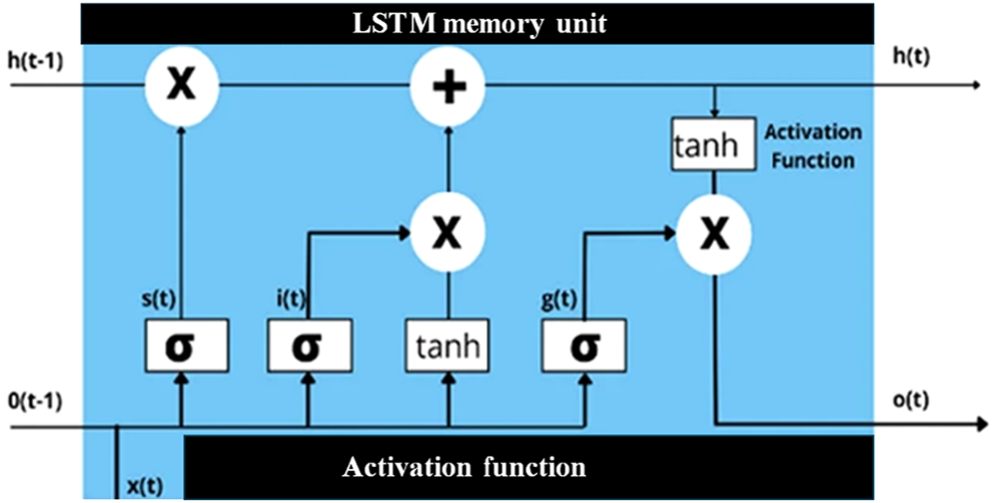
Structure of an LSTM cell with a memory unit and nonlinear gates.

Forget gate (*g*_*t*_): Selectively removes irrelevant information from the previous cell state *S_t_*_–1_ through elementwise multiplication, ensuring that outdated or noninformative process features do not interfere with ongoing monitoring. Input gate (*i*_*t*_): Regulates the degree to which new information is written into the memory cell. A candidate cell state is generated and modulated to determine what aspects of the new input are useful for identifying faults. Cell state update (*S_t_*): Combines the retained memory and new insights to form an updated representation of the system’s state. Output gate (*o*_*t*_): It determines which components of the updated cell state contribute to the current output *h*_*t*_ for fault classification. The sigmoid function is mathematically expressed as

7 $$\mathrm{sigmoid}\left(x_{i}\right)=\frac{1}{1+e^{-x_{t}}}$$

The LSTM architecture is expressed as follows

8 $$g_{t}=sigmoid\left(w_{g}\times \left[h_{\left(t-1\right)}x_{t}\right]+b_{g}\right)$$

9 $$i_{t}=\mathrm{sigmoid}\left(w_{i}\times \left[h_{\left(t-1\right)}x_{t}\right]+b_{i}\right)$$

10 $$o_{t}=\mathrm{sigmoid}\left(w_{o}\times \left[h_{\left(t-1\right)}x_{t}\right]+b_{0}\right)$$

11 $${\mathrm{S̃}}_{t}=\tanh\left(w_{s}\times \left[h_{\left(t-1\right)}\right]+b_{s}\right)$$

12 $$S_{t}=g_{t}⊙S_{\left(t-1\right)}+i_{t}⊙{\mathrm{S̃}}_{t}$$

13 $$h_{t}=o_{t}⊙\tan\left(S_{t}\right)$$

where *x*_*t*_ = input vector at time step *t*. *w*_*g*_, *w*_*i*_, *w*_*o*_, and *w*_*s*_ are weight matrices for each gate. *b*_*g*_, *b*_*i*_, *b*_0_, and *b*_*s*_ are bias terms. ⊙ = element-wise multiplication. *S̃*_*t*_ = candidate cell state.

#### 3.2.3 Activation Function

Activation functions are essential in deep learning, enabling neural networks to capture nonlinear patterns in dynamic, multisensor industrial data.^54^ By introducing nonlinearity, they enhance the learning capacity and diagnostic accuracy, allowing effective discrimination between normal and faulty states. Applied at each layer, they transform weighted inputs during forward propagation and support gradient-based weight updates during back propagation, which is critical for detecting incipient or slowly evolving faults. For ReLU, positive inputs are retained while negative values are set to zero (eq 14).

14 ReLu(x)=max(0,x)

During backpropagation, ReLU functions as an integral layer in the deep learning architecture, with its output determined by the layer’s position in the network. Let *x* ^(*l*)^ and *x* ^(*l*+1)^ represent the outputs of the (*l*)-th and (*l* + 1)-th layers. The corresponding partial derivatives of the loss function with respect to these outputs are derived using eq 15.

15 $$\delta^{L}=\frac{\delta L}{{\delta x}^{l}}=\frac{\delta L}{{\delta x}^{\left(l+1\right)}}\times \frac{{\delta x}^{\left(l+1\right)}}{{\delta x}^{l}}=\delta^{\left(l+1\right)}\times \frac{\delta ReLu\left(x^{l}\right)}{{\delta x}^{l}}$$

where *x* = output, *L* = loss function.

As illustrated in Figure 4, activation functions such as linear, ReLU, sigmoid, and tanh strongly affect the model convergence and performance. Sigmoid is typically used for probabilistic outputs, while ReLU and its variants (e.g., Leaky ReLU) provide faster convergence and alleviate vanishing gradient issues.^52^

**Figure 4:**
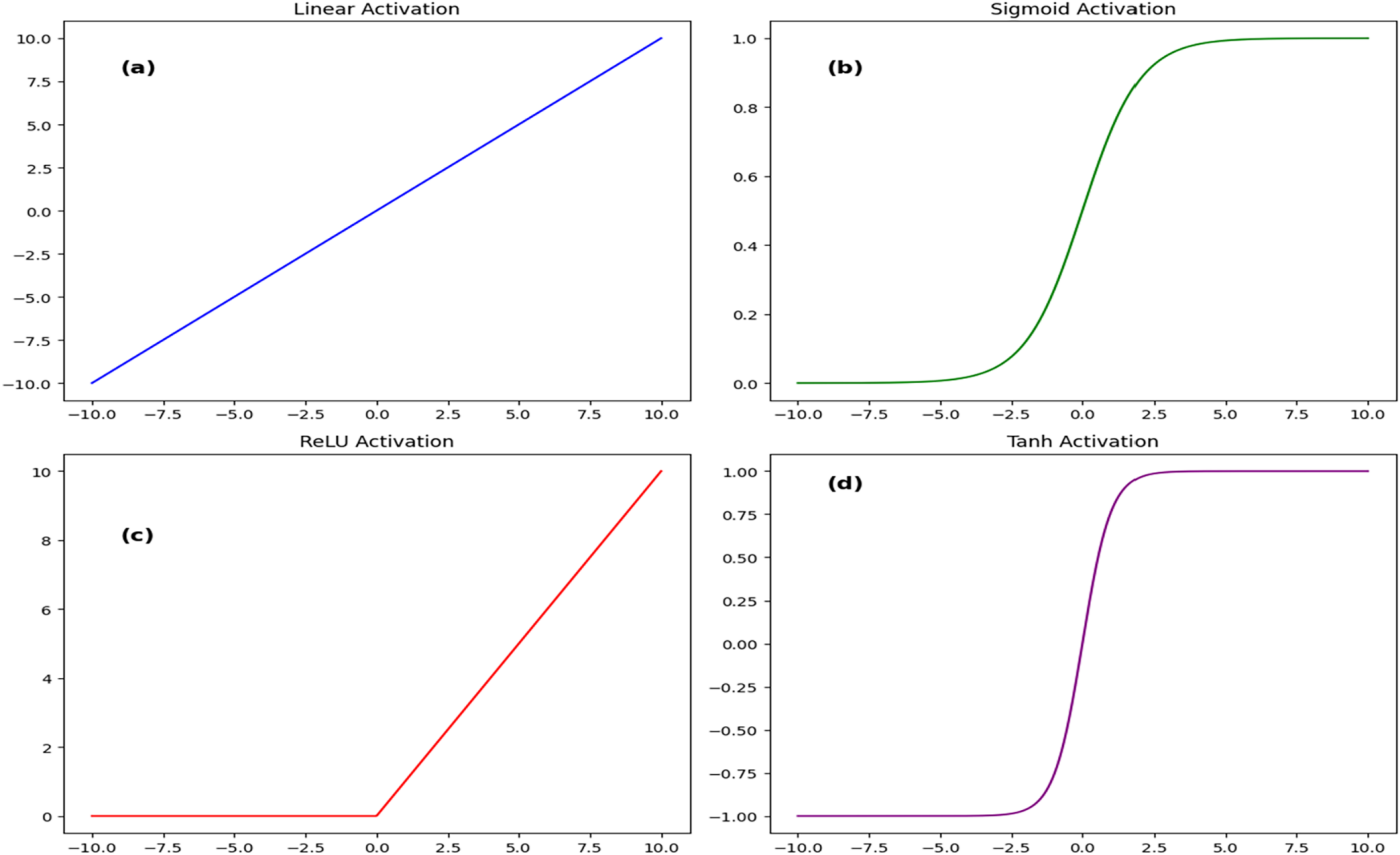
Popular activation functions: (a) linear; (b) sigmoid, (c) ReLU, and (d) tanh.

The primary role of an activation function is to transform aggregated inputs into meaningful outputs for subsequent computations.^54^

#### 3.2.4 Adam Optimizer

The Adam (adaptive moment estimation) optimizer is a widely used deep learning algorithm that combines adaptive learning rates with momentum-based updates. By integrating the strengths of momentum and RMSProp, Adam overcomes limitations of earlier methods such as SGD and AdaGrad, enabling efficient and robust convergence in nonconvex optimization problems.^55^ Unlike SGD, Adam adaptively tunes parameter-wise learning rates using exponentially decaying averages of first- and second-order gradient moments, allowing effective handling of noisy or sparse gradients common in industrial fault diagnosis. The Adam optimizer is widely adopted in deep learning training because of its ability to efficiently handle complex gradients and dynamic loss landscapes. In chemical process monitoring, it promotes stable and faster convergence, enhancing the model reliability. The Adam optimizer equations are given as follows:

For the gradient

16 $$g_{t}=\nabla_{\theta }J\left(\theta_{t}\right)$$

First-moment estimate or mean equation

17 $$m_{t}=\beta_{1}\times m_{\left(t-1\right)}+\left(1-\beta_{1}\right)\times g_{t}$$

Second-moment estimate equation

18 $$v_{t}=\beta_{2}\times v_{\left(t-1\right)}+\left(1-\beta_{2}\right)\times g_{t}^{2}$$

The equation for bias correction

19 $${\mathrm{m̃}}_{t}=\frac{m_{t}}{\left(1-\beta_{1}^{t}\right)},v_{t}=\frac{v_{t}}{\left(1-\beta_{2}^{t}\right)}$$

The equation for updating parameters

20 $$\theta_{\left(t+1\right)}=\theta_{t}-\alpha \times \frac{{\mathrm{m̃}}_{t}}{\sqrt{{ṽ}_{t}+\varepsilon }}$$

where *g*_*t*_ is the gradient of the objective at time step *t*. θ = the model parameters. *m*_*t*_ is the mean of past gradients with exponential decay (first moment). *v*_*t*_ = exponentially weighted moving average of past squared gradients (second moment). *m̃*_*t*_, *ṽ*_*t*_ = the estimates of bias correction. β_1_, β_2_ = the moment estimates decay rates. α = the learning rate. ε = a small constant added to ensure numerical stability.

The optimizer’s default parameters are α = 0.001, β_1_ = 0.9, β_2_ = 0.999, and ε = 10^–8^.

### 3.3 Dempster–Shafer Theory

Dempster–Shafer theory (DST) is a mathematical framework for handling uncertainty and incomplete information, introduced by Dempster and extended by Shafer.^56^ Unlike classical probability, DST represents partial beliefs using belief functions, making it effective for noisy or unreliable sensor data. In process monitoring, where sensors may fail or provide inconsistent readings, DST’s strength lies in combining evidence from multiple independent sources through Dempster’s rule of combination. This enables robust multisensor fusion and supports accurate fault diagnosis under uncertainty.

#### 3.3.1 Basic Mathematical Concepts of DST

At the core of DST is the frame of discernment (Θ), which represents the complete set of mutually exclusive hypotheses regarding a system’s state, such as normal operation and fault conditions. DST assigns belief not just to individual hypotheses but also to subsets of the frame, enabling a more flexible and expressive representation of uncertainty.^57^ The key mathematical terms include the basic probability assignment (BPA), also known as the mass function (*m*), which assigns belief to each subset of Θ, as expressed in the equations below

21 m:2Θ→[0,1]

22 m(Θ)=0

23 $$\{\begin{array}{l} m\left(\Theta \right)=0\\ \sum_{R\subseteq \Theta }m\left(R\right)=1 \end{array}$$

Belief function (Bel): Represents the total belief supporting a subset Θ. The mass *m*(*A*) quantifies the probability assigned specifically to *A*, isolating evidence uniquely supporting that fault condition. Assigning mass functions over the power set allows defining a probability interval for any event, bounded by the degree of belief Bel­(*A*), which sums the mass of all nonempty subsets in *A*, and the degree of plausibility Pl­(*A*), which reflects how evidence does not contradict *A*. These bounds enable reasoning under uncertainty, crucial for fault detection with incomplete information.

24 $$\mathrm{Bel}\left(A\right)=\sum_{B\subseteq A}m\left(B\right)$$

Plausibility function (Pl) reflects the maximum amount of belief that could support *A*.

25 $$\mathrm{Pl}\left(A\right)=1-\mathrm{Bel}\left(A\right)=\sum_{B\cap R\neq 0}m\left(B\right)$$

The belief function and the plausibility function are connected through the following relationship

26 Pl(A)=1−Bel(Ã)

For a given subset *A*, Bel­(*A*) and Pl­(*A*) denote the lower and upper bounds of the probability interval, respectively. The range Bel­(*A*), Pl­(*A*) represents the degree of uncertainty.

#### 3.3.2 Dempster’s Combination Rule

A combination rule is employed to integrate two independent mass functions *m*_1_ and *m*_2_ into a new mass function *m*, considering only consistent evidence over the frame of discernment Θ, as expressed in eqs 27–29.

27 m=m⊕m⊕...⊕m(n−1times)

28 $$m\left(A\right)=\{\begin{array}{l} \frac{\sum_{A\cap B=0}m_{1}\left(A\right)m_{2}\left(B\right)}{\left(1-K\right)},A\neq \Theta \\ 0,A=\Theta \end{array}$$

29 $$K=\sum_{A\cap B=\Theta }m_{1}\left(A\right)m_{2}\left(B\right)$$

where *K* is the conflict coefficient, representing the total conflicting mass. Equation 29 applies a normalization factor, 1 – *K*, to account for consistency among multiple sources of evidence while disregarding conflicts by assigning the conflicting mass to the null set. These principles enable DST to integrate uncertain or inconsistent evidence. Dempster’s rule redistributes conflict mass among consistent hypotheses, preventing contradictory evidence from dominating the decision (Table S1). For example, consider two temperature sensors monitoring a distillation column for potential abnormalities: Sensor 1 assigns belief masses as

$$m_{1}\left(\{F_{A}\}\right)=0.65,m_{1}\left(\{F_{B}\}\right)=0.25,\;\mathrm{and}\;m_{1}\left(\{F_{A}\cup F_{B}\}\right)=0.10$$

Sensor 2 provides

$$m_{2}\left(\{F_{A}\}\right)=0.55,m_{2}\left(\{F_{B}\}\right)=0.35,\;\mathrm{and}\;m_{2}\left(\{F_{A}\cup F_{B}\}\right)=0.10$$

Using Dempster’s combination rule, the two sets of evidence are fused to generate a single, more reliable estimate of the fault condition (either *F*_*A*_ or *F*_*B*_).

#### 3.3.3 Key Advantages of DST

Dempster–Shafer theory provides flexibility for developing customizable fault diagnosis frameworks in complex industrial processes. It imposes no strict rules on constructing or combining mass functions, which can range from simple to complex and be iteratively updated based on process dynamics. A major advantage is its ability to model uncertainty without prior probabilities, enabling the detection of unseen faults. DST also represents ignorance through mass values, reflecting diagnostic confidence, an essential feature for handling incomplete or noisy sensor data. This adaptability makes DST well-suited for process-specific fault detection systems.

## 4 Results and Discussion

While Dempster–Shafer theory is highly effective in representing uncertain and conflicting evidence, it cannot automatically extract high-level features from raw sensor data, a task at which DL excels. Conversely, DL models can learn complex patterns from high-dimensional inputs and are not inherently equipped to handle uncertainty. Both approaches also lack interpretability. To overcome these complementary limitations, this study introduces a novel framework that integrates DL’s feature extraction capabilities with DST’s uncertainty modeling and feature attention for enhanced interpretability. In this framework, DL extracts meaningful features from sensor inputs, while DST fuses classification results to capture uncertainty and reinforce consistent evidence, with attention mechanisms highlighting fault-relevant features (Figure 5).

**Figure 5:**
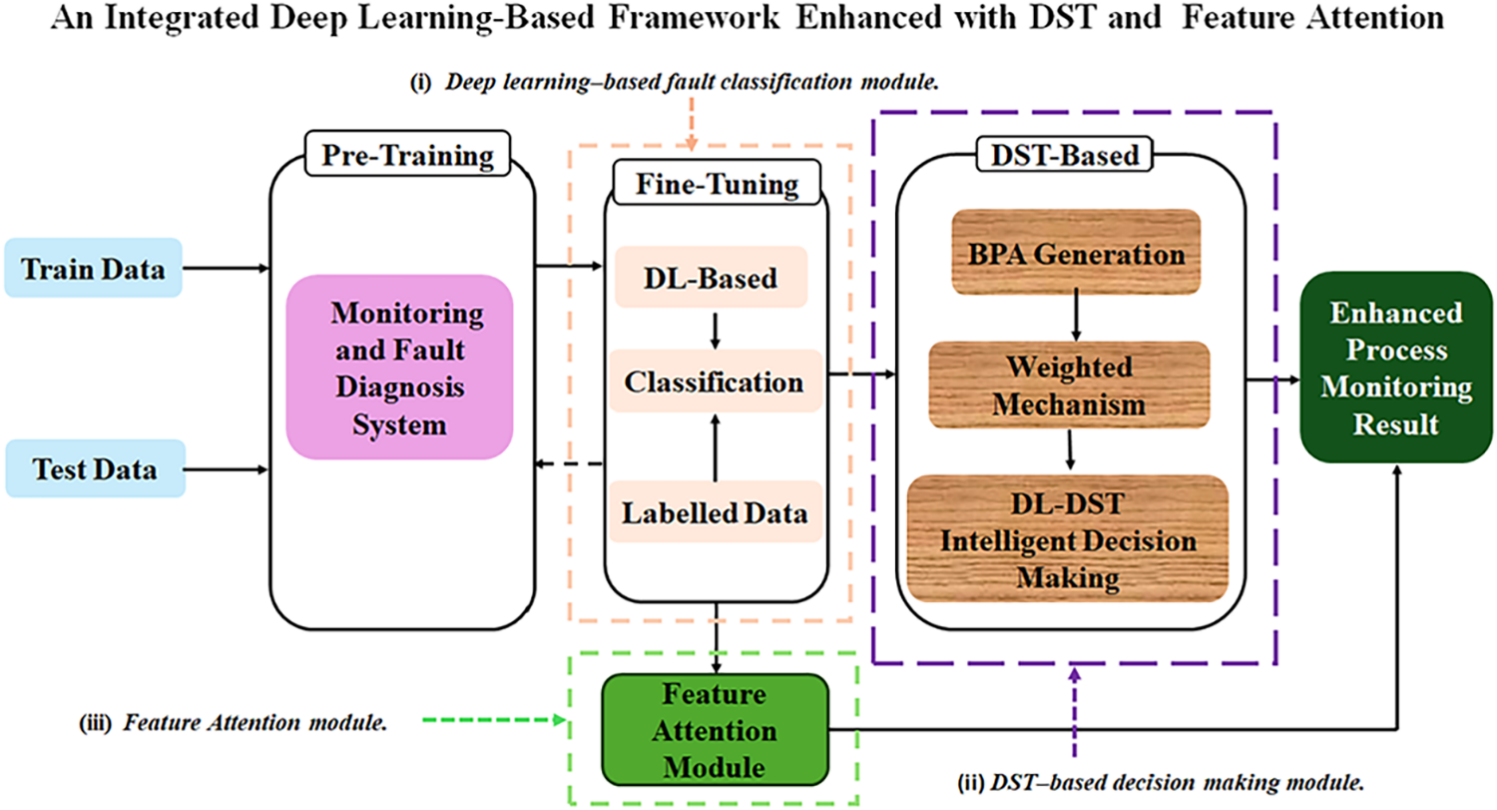
Basic structure of the integrated framework.

Raw process data, often affected by noise, is first processed through the DL module to extract spatial and temporal features while reducing dimensionality. An attention mechanism selectively weights fault-relevant features, enhancing interpretability and sensitivity to critical process variables. The class-wise outputs of the DL model are transformed into DST belief mass functions, with each fault category weighted by classification accuracy. Dempster’s rule aggregates these mass functions, resolving conflicts and minimizing uncertainty across sensors. This integration enables reliable multiclass fault diagnosis even with noisy or conflicting data. The DL module abstracts complex process patterns and supports high-dimensional feature representation, while the DST fusion enhances robustness and consistency. Attention further emphasizes informative features, improving sensitivity and interpretability. Together, the framework provides a fault-resilient, adaptive monitoring system, validated using the Tennessee Eastman benchmark process.

### 4.1 Feature Extraction Using Deep Learning Models

At this stage, two deep learning networks are constructed: an autoencoder for spatial feature extraction and a long–short-term memory (LSTM) network for temporal pattern learning. The autoencoder, composed of balanced encoder and decoder layers, compresses the high-dimensional sensor inputs into a compact latent space and reconstructs them, thereby capturing the underlying spatial relationships among variables (Figure 6). Meanwhile, the LSTM, utilizing memory cells with input, output, and forget gates, is responsible for modeling long-range dependencies and evolving temporal behaviors in process data. The autoencoder is trained exclusively on normal, unlabeled operating data to learn representative spatial feature embeddings.

**Figure 6:**
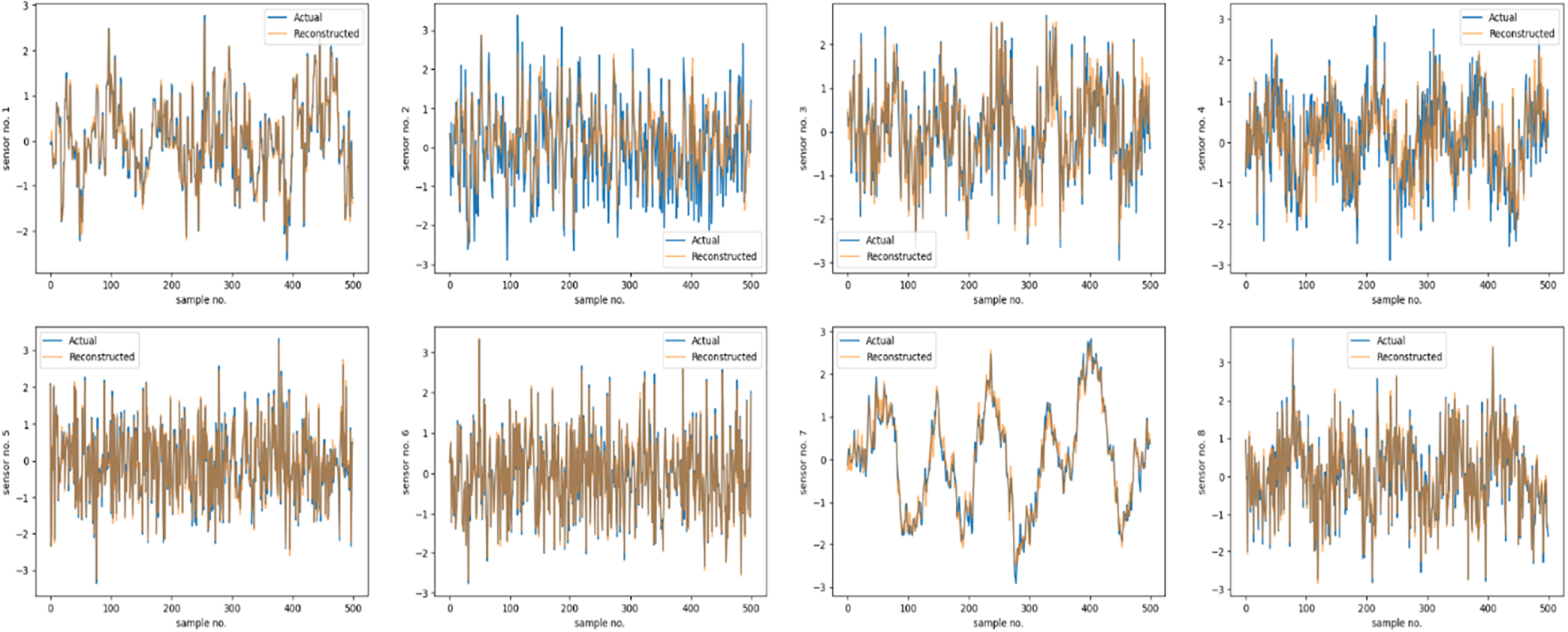
Feature extraction using the deep learning model.

The LSTM is trained on labeled normal and fault conditions to learn temporal fault patterns using categorical cross-entropy loss and Adam optimization. The LSTM and autoencoder progressively improve in feature extraction, classification, and generalization, effectively distinguishing normal states from faulty states.

#### 4.1.1 Construction of an Autoencoder Model

The proposed architecture adopts a noise-robust autoencoder framework that is aimed at extracting discriminative features from multivariate process measurements. The encoding stage begins by accepting normalized sensor inputs to facilitate effective feature representation learning. To strengthen robustness against measurement disturbances and improve generalization, Gaussian noise with a standard deviation of 0.05 is injected into the input layer, as summarized in Table 2. This noise injection functions both as a denoising mechanism and as a form of regularization.

**Table 2:** Parameters of the Autoencoder Model

parameter	value	description
encoder layer 1	128	learns sparse, nonlinear feature representations.
encoder layer 2	64	compresses features to a lower-dimensional latent space.
decoder layer	64	begins reconstruction from latent features.
output layer	linear	reconstructs the original continuous input values.
weight decay	1 × 10^–6^	regularizes weights to prevent overfitting.
noise injection	0.05	improves robustness to noise and acts as a regularizer.
loss function	MSE	measures the reconstruction accuracy of normal data.
dropout	0.3	prevents overfitting by randomly deactivating neurons.
optimizer	Adam	adaptive optimization algorithm for efficient convergence.
learning rate	0.001	controls the step size during weight updates.
batch size	256	how many samples are used per training step.
epochs	200	number of training iterations.

The perturbed inputs are subsequently processed by a dense layer (128 neurons) with ReLU activation, enabling complex mapping and promoting compact, informative feature representations. To support stable and accelerated training, batch normalization is applied immediately after the activation. This normalizes layer outputs across mini-batches and reduces sensitivity to initial weight values. A dropout layer with a rate of 0.3 is subsequently applied to randomly deactivate neurons during training, helping to prevent overfitting and encourage the learning of more generalized features. A subsequent dense layer containing 64 units with ReLU activation further reduces the feature space, thereby finalizing the encoder architecture. The decoder reconstructs the original input, starting with dense layers of 64 and 128 neurons with ReLU activation and batch normalization, followed by a linear output layer. The model is optimized using Adam (learning rate 0.001, weight decay 10^–6^) to minimize mean squared error (MSE) between inputs and reconstructions. Early stopping (30 stagnant epochs) and a learning rate scheduler from the 10th epoch ensure stable and efficient convergence for fault detection.

#### 4.1.2 Construction of the LSTM Model

The model is constructed using a two-layer stacked long–short-term memory (LSTM) network designed for sequential sensor data classification. This design efficiently captures temporal dependencies and patterns across the time-series data. The first LSTM layer has 128 units and utilizes a sigmoid function to capture nonlinear time patterns. It returns the full sequence so that later layers maintain the time structure. Immediately afterward, batch normalization stabilizes the activations and minimizes changes in the input data during training, improving both learning and convergence. A second LSTM layer with 128 units and a sigmoid activation condenses the sequence into a fixed-size vector that captures the timing patterns in the input. To promote generalization and prevent overfitting, dropout regularization was applied with a rate of 0.4. This stochastic regularization method deactivates a portion of the neurons during each training step, reducing the dependency on specific features. The output passes through two dense layers: 300 ReLU neurons with dropout (0.4) followed by a SoftMax layer for multiclass probabilities. The network is optimized using Adam (learning rate = 0.0001) and categorical cross-entropy on normalized 3D inputs (epochs = 200; batch size = 64), demonstrating stable convergence and reliable generalization, as summarized in Table 3.

**Table 3:** LSTM Model Hyperparameters

hyperparameters	value	description
first LSTM layer	128	captures nonlinear temporal patterns and returns the full sequence.
second LSTM layer	128	encodes the temporal sequence into a fixed-length feature vector.
dense layer	300	transforms temporal features into higher-level representations.
output layer	SoftMax activation	produces class probabilities for multiclass fault classification.
loss function	categorical cross-entropy	evaluates how closely the predicted fault categories match the true fault labels.
dropout	0.4	regularization is applied to prevent overfitting after LSTM layers.
optimizer	Adam	ensures efficient and stable training.
learning rate	0.0001	balances convergence speed and gradient stability.
batch size	64	batch size for each training cycle.
epochs	200	total training steps needed for convergence.

### 4.2 Implementation Procedure of the Integrated Framework with DST

Multisource evidence is fused using an improved DST, converting deep learning confidence scores into BPAs weighted by model accuracy. Weighted averaging manages conflicts and preserves redundant evidence, enhancing DL–DST fault diagnosis reliability in three steps (Figure 7).

**Figure 7:**
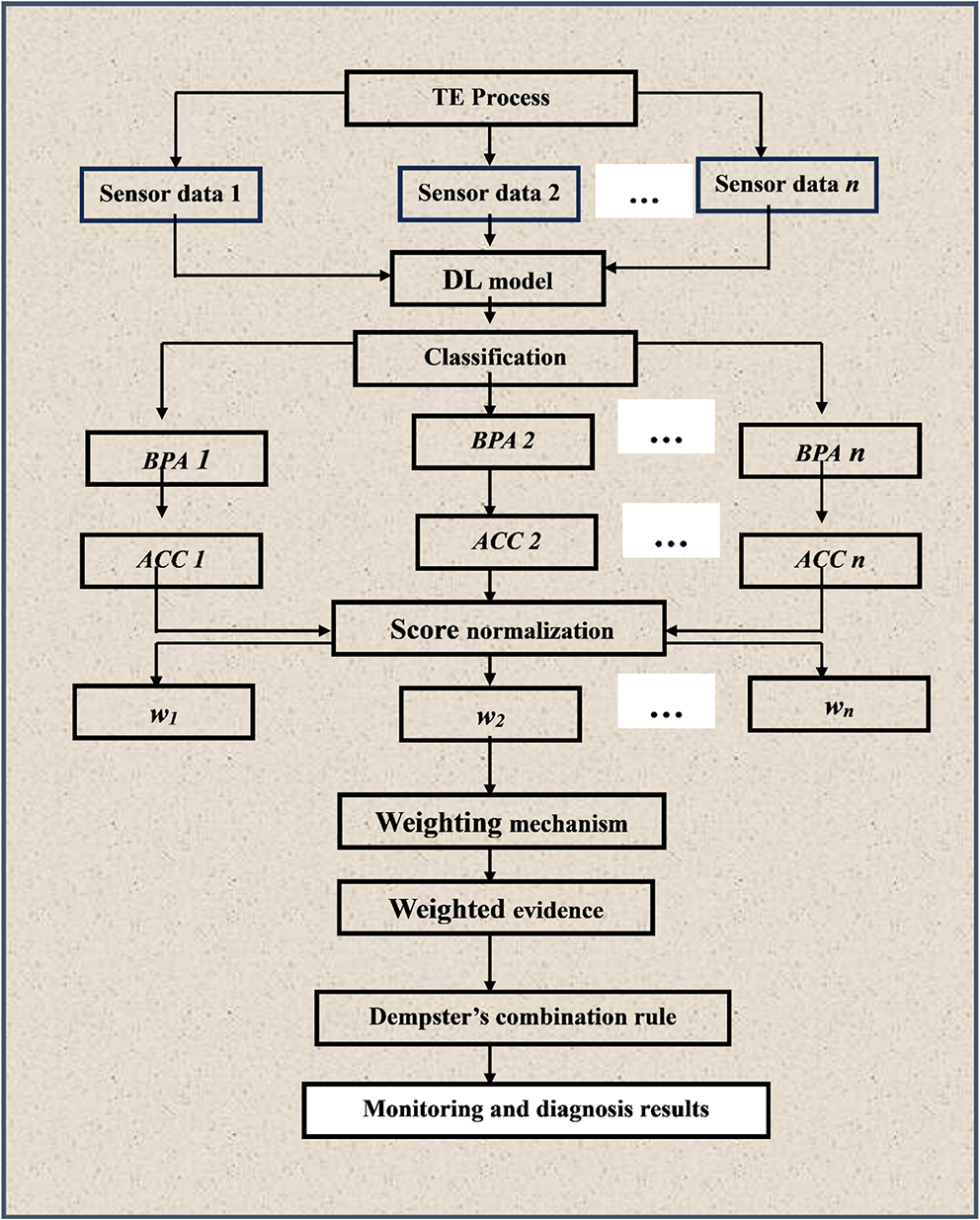
Implementation procedure of the framework with DST integration.

#### 4.2.1 Step 1: Autoencoder Training on Multiclass Data

Process sensor data from normal and faulty conditions are input to the autoencoder, which compresses and reconstructs it to reveal hidden patterns. Trained with Adam to minimize MSE, the encoder extracts key features, while the decoder reconstructs inputs. Normal data are well-reconstructed, whereas faults yield higher errors, enabling detection and supporting classification, with validation ensuring generalization.

#### 4.2.2 Step 2: Fine-Tuning the Autoencoder Model

After pretraining, the autoencoder is fine-tuned with labeled data, adding ReLU in the final layers for fault classification. Pretrained weights are retained, and the network is trained end-to-end using backpropagation. MSE remains the main loss, with dropout and batch normalization preventing overfitting. Fine-tuning improves fault detection, classification, and generalization under unseen conditions.

#### 4.2.3 Step 3: Integration of Evidence from Multiclass Classification Using DST

The trained autoencoder produces confidence scores converted into BPAs, weighted by model reliability, and combined to yield robust multiclass fault diagnoses. Each deep learning model, including LSTM, is trained independently. Algorithm 1 presents the corresponding pseudocode for clarity.
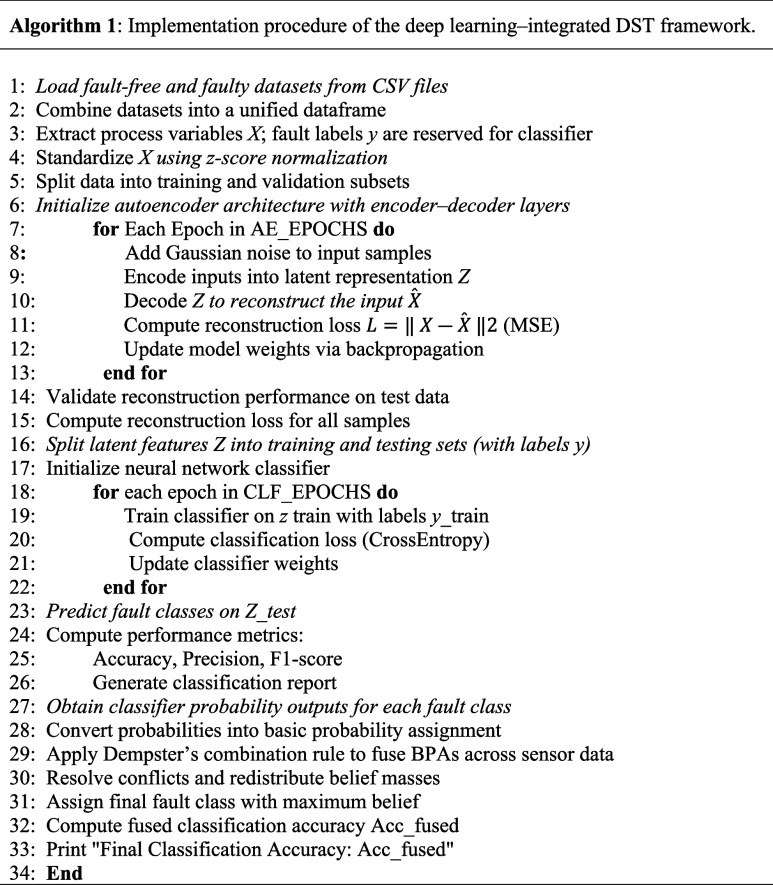


### 4.3 Implementation Procedure of the Integrated Feature-Attention Framework

As shown in Figure 8, the framework uses a dual-branch, feature-attention deep learning architecture for multiclass fault classification. Preprocessed and normalized process measurements are fed into two complementary branches that capture the spatial and temporal fault patterns.

**Figure 8:**
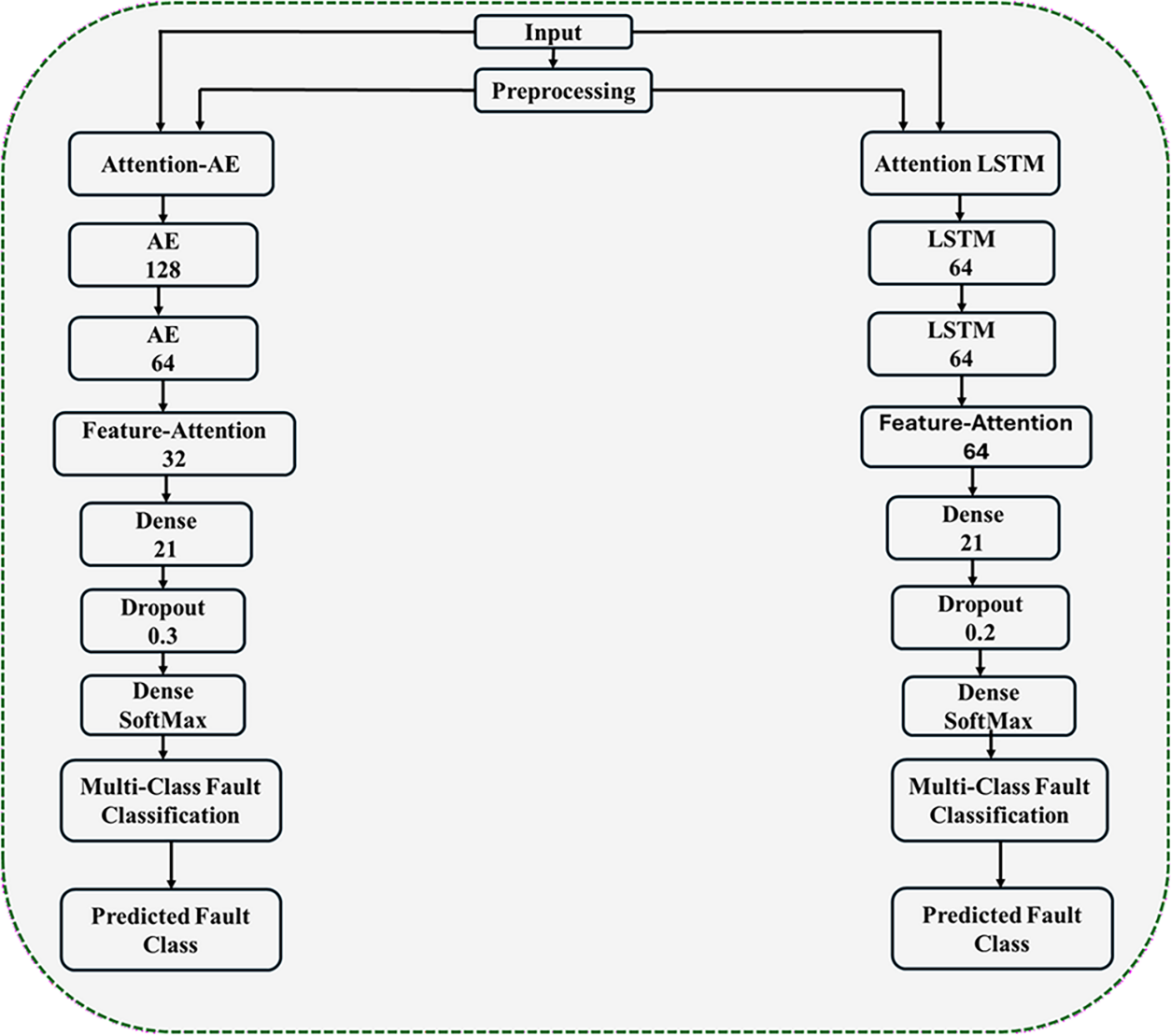
Implementation procedure of the integrated feature-attention framework.

#### 4.3.1 Step 1: Feature Extraction

The Attention-AE branch extracts nonlinear spatial correlations among process variables via stacked autoencoders, while the Attention-LSTM branch captures temporal fault patterns by using an attention-enhanced LSTM network.

#### 4.3.2 Step 2: Feature-Attention Mechanisms

Feature-attention modules in both branches adaptively weight important variables, highlighting critical spatial features in AE and key temporal features in LSTM, enhancing interpretability and diagnostic accuracy.

#### 4.3.3 Step 3: Multiclass Classification with Regularization

Attention-refined features pass through dense layers with dropout (0.3 for AE and 0.2 for LSTM) and SoftMax outputs to predict fault classes independently while reducing overfitting.

#### 4.3.4 Step 4: Training Strategy

Early stopping and learning rate reduction enhance the generalization. The dual-branch, feature-attention architecture ensures accurate, interpretable, and robust multiclass fault diagnosis.

### 4.4 Experimentation and Model Evaluation

#### 4.4.1 Tennessee Eastman Process

The Tennessee Eastman (TE) process, often referred to as TEP, was developed by Downs and Vogel in 1993 at Eastman Chemical Company in Tennessee, USA.^58^ It serves as a dynamic simulation of a realistic chemical production system, providing a complex yet controlled environment for research in process control, monitoring, and fault diagnosis. The TE process has gained broad acceptance in academia and industry for evaluating the performance of data-driven and model-based diagnostic frameworks. The process comprises five primary units: a reactor, condenser, compressor, separator, and stripper (Figure 9). It involves complex chemical reactions where four gaseous reactants (A, C, D, and E) are fed into a reactor along with an inert component (B). The reactor facilitates the formation of two liquid products (G and H) while also generating a liquid byproduct (F). These reactions are irreversible and exothermic and follow approximately first-order kinetics with respect to reactant concentrations. The reaction pathways include

30 A(g)+C(g)+D(g)→G(l)

31 A(g)+C(g)+E(g)→H(l)

32 A(g)+E(g)→F(l)

33 3D(g)→2F(l)

**Figure 9:**
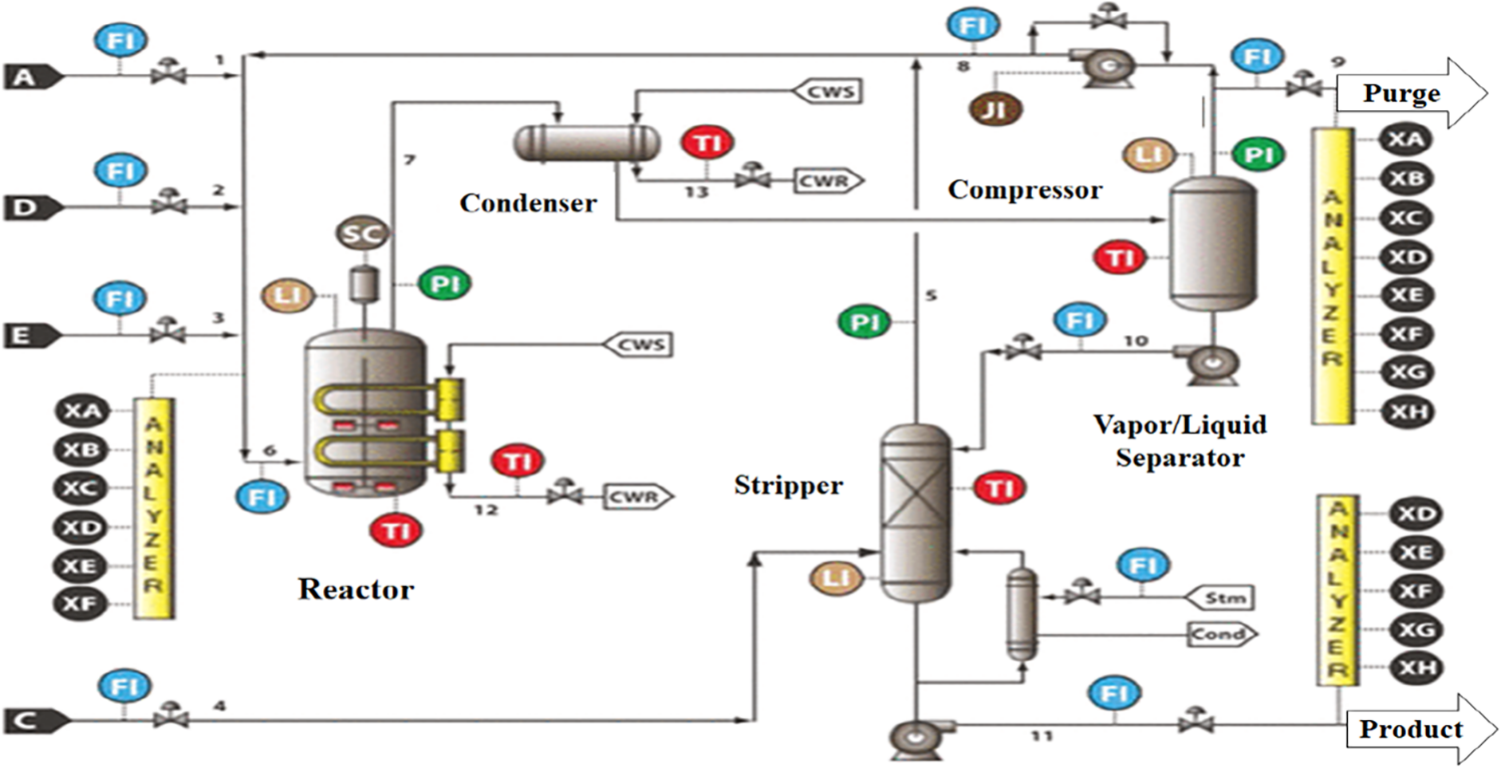
Flow diagram of the Tennessee Eastman process.

After leaving the reactor, the product stream is condensed and separated into the vapor and liquid phases. The vapor is compressed and recycled to the reactor, with a purge to prevent inert and byproduct accumulation, while the liquid stream enters a stripper to remove residual reactants, yielding products G and H for downstream processing.

#### 4.4.2 Tennessee Eastman Process Variables

The Tennessee Eastman process is characterized by 52 key variables: 41 measured and 11 manipulated. Nineteen of the measured variables are associated with composition analysis, while Table 4 details how the manipulated variables control the various process operations.

**Table 4:** Manipulated Control Variables

manipulated variables	process description
XMV(1)	D feed flow stream 2
XMV(2)	E feed flow stream 3
XMV(3)	A feed flow stream 1
XMV(4)	total feed flow stream 4
XMV(5)	compressor recycle valve
XMV(6)	purge valve
XMV(7)	separator product liquid flow
XMV(8)	stripper product liquid flow
XMV(9)	stripper steam valve
XMV(10)	reactor cooling water flow
XMV(11)	condenser cooling water flow

The measured variables, detailed in Table S2, provide real-time feedback on system performance. Specifically, input variables numbered 1–36 correspond to process measurements, and inputs 1–11 represent the manipulated control variables. The output variables, which reflect product quality metrics, are labeled from 37 to 41. The Tennessee Eastman process simulation defines a total of 20 distinct fault types in addition to a normal operating condition, as presented in Table 5. Among these, 15 are known faults with identifiable causes comprising step changes (Faults 1–7), random variations (Faults 8–11), a slow drift fault (Faults 12–13), and valve sticking faults (Faults 14–15). The last five faults (16–20) are treated as unknown faults due to unspecified causes, making detection challenging. Each fault has corresponding training and testing datasets obtained from 24-h and 48-h simulations, with set-point changes introduced at *t* = 30. The integrated deep learning model is trained on these datasets and tested for its ability to correctly identify all 20 faults using the TEP benchmark.

**Table 5:** Distinct Fault Types in the TE Process

fault ID	fault descriptions	fault class
F1	A/D feed ratio changes, B composition constant	step
F2	B feed ratio changes, A/D composition constant	step
F3	D feed temperature changes	step
F4	reactor cooling water inlet temperature changes	step
F5	condenser cooling water inlet temperature changes	step
F6	A feed loss	step
F7	C head pressure loss	step
F8	A/B/C composition changes	random variation
F9	D feed temperature changes	random variation
F10	C feed temperature changes	random variation
F11	reactor cooling water inlet temperature changes	random variation
F12	separator cooling water inlet temperature changes	slow drift
F13	reactor dynamic constant changes	slow drift
F14	reactor valve	sticking
F15	separator valve	sticking
F16	unknown	unknown
F17	unknown	unknown
F18	unknown	unknown
F19	unknown	unknown
F20	unknown	unknown

#### 4.4.3 Data Collection and Preprocessing

To support the training and evaluation of fault diagnosis models, datasets were generated under both normal and faulty conditions using the Tennessee Eastman process simulator. The training datasets consist exclusively of normal (fault-free) data, with 480 time samples recorded for each fault condition. A time-lag window of 40 samples is applied to capture the temporal dynamics of the process. A total of 21 training datasets are used, one for normal conditions and 20 for various fault conditions. The testing datasets include both normal and faulty conditions, with each dataset consisting of 960 samples. In each testing dataset, faults are introduced starting at the 160th sample. Moreover, dynamic augmentation of the data is implemented to increase test set robustness, promoting better generalization to unknown fault situations. *Z*-score normalization centers each variable to zero mean and unit variance, preventing dominance by larger-range features and ensuring balanced contributions during model training and evaluation (eq 34).

34 $$x_{\mathrm{std}}=\frac{x-\mu }{\sigma }$$

where *x*_std_ is the normalized value, σ represents the feature’s standard deviation, *x* denotes the original feature value, and μ is the feature’s mean.

### 4.5 Integrated Framework Implementation and Performance Evaluation

The proposed integrated framework was implemented using Keras on the TensorFlow platform due to its simplicity and computational efficiency.^59^ Simulations were conducted on a 64-bit Windows 10 system equipped with an Intel Xeon E5–2643 CPU, 8 GB of RAM, and an AMD FirePro V5900 GPU. GPU-accelerated training was performed by using the Keras functional API. The dataset was divided into training, validation, and testing subsets to ensure an unbiased model evaluation. An 80:20 split was used for training and validation, while separate testing datasets were used for performance assessment. The model was trained using the Adam optimizer with optimized hyperparameters.

#### 4.5.1 Performance Metrics

Accurate performance evaluation in multiclass fault diagnosis requires robust metrics that capture both detection and classification capability. Accordingly, the proposed models are evaluated using accuracy, precision, and F1-score, which collectively assess classification correctness and balanced sensitivity across fault classes in complex chemical processes.^34,60^ Among these, accuracy was chosen as the primary metric to monitor the learning capability of the model across different experimental setups. It offers a straightforward measure of overall correctness in classification, making it suitable for evaluating performance across multiclass fault diagnosis scenarios. The values for accuracy, precision, and F1-score were computed using standard formulas outlined in eqs 35–37, following the methodology proposed in ref 61. These metrics collectively ensure accurate, robust, and consistent fault detection and classification in process monitoring.

35 $$accuracy=\frac{TP+TN}{TP+FN+TN+FP}$$

36 $$precision=\frac{TP}{TP+FP}$$

37 $$F1‐score=\frac{\left(2\times TP\right)}{\left(2\times TP+FN+FP\right)}$$

where TP = true positives, TN = true negative, FP = false positives, and FN = false-negative.

#### 4.5.2 Training and Testing Sets

A symmetric encoder–decoder autoencoder is employed for unsupervised fault detection. The encoder processes normalized sensor data with added Gaussian noise for robustness and comprises two dense layers (128 and 64 units, ReLU), followed by batch normalization and dropout (0.3). The decoder mirrors this structure with dense layers of 64 and 128 units (ReLU) and a linear output layer for input reconstruction. The model is trained for 200 epochs with a batch size of 256 using the Adam optimizer (learning rate 0.001, decay 10^–6^) and MSE loss. Early stopping and learning rate scheduling are applied to enhance generalization, enabling reliable detection of deviations from normal operation (Figure 10).

**Figure 10:**
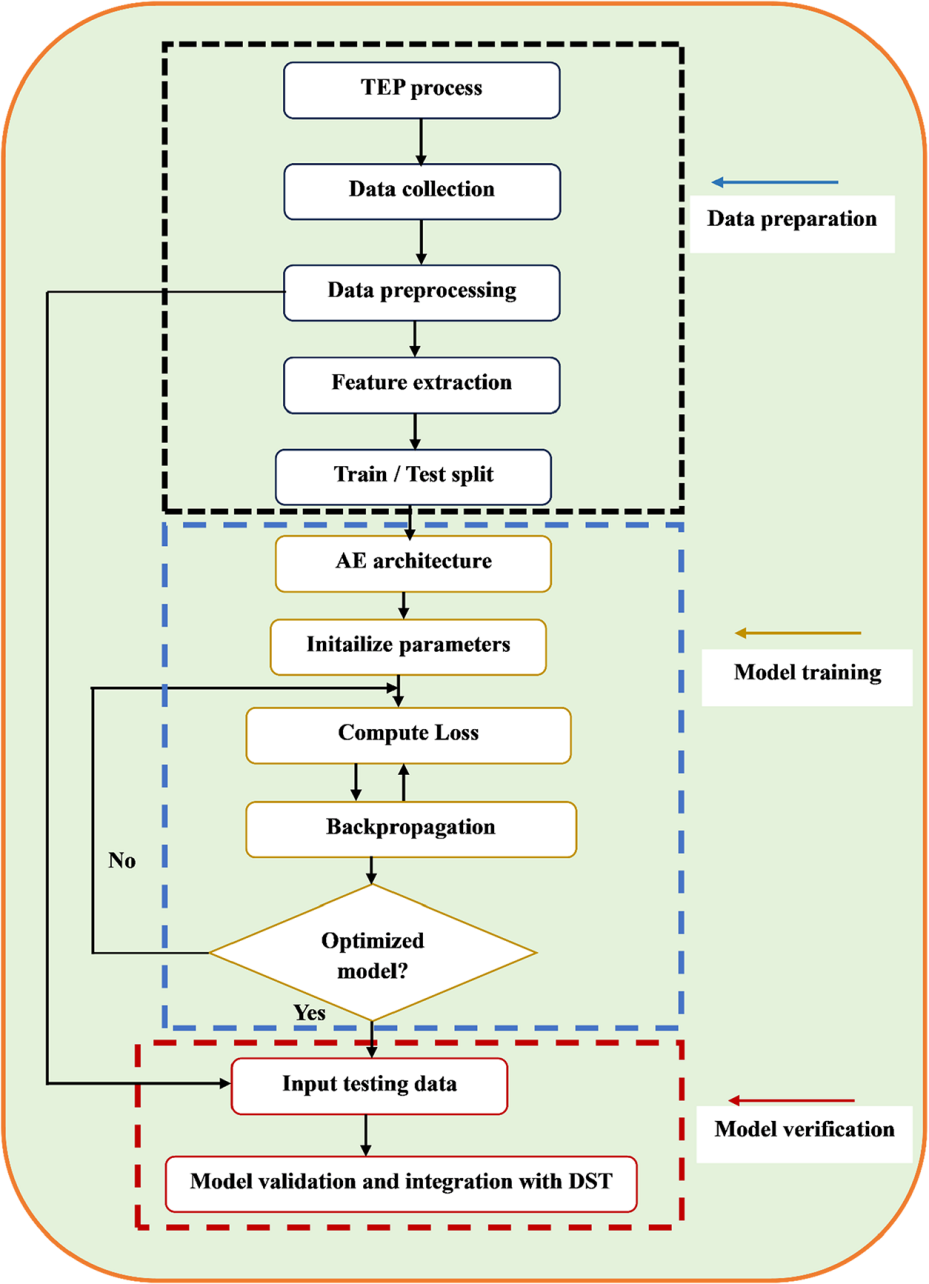
Training an autoencoder model to enable validation and integration.

### 4.6 Optimizing Autoencoder Model Hyperparameters

Hyperparameter tuning was performed to optimize the AE model for the TEP. A two-step approach combined an initial manual search, guided by prior knowledge,^62,63^ with a grid search to identify optimal learning rates (0.00001–0.01), epochs (100–400), dropout rates (0.1–0.4), and layer counts (2–5). The best configuration was chosen based on maximum validation accuracy (Tables S3–S6). Final evaluation on a stratified test set used class-balanced metrics, confirming the AE’s effectiveness in modeling normal behavior and detecting faults.

#### 4.6.1 Effect of Layer Depth on the Performance of the Autoencoder Model

Autoencoder depth is a vital factor for learning intricate data patterns. Here, we investigated the impact of the number of layers on the AE model’s fault detection performance in the Tennessee Eastman process. Figure 11 illustrates how increasing layers from 2 to 5 affects accuracy, precision, and F1-score. For configurations with 2–4 layers, the AE model consistently achieved a high precision of 88%, indicating reliable detection of true fault conditions while keeping false alarms low. Accuracy also remained stable at 85%, indicating that overall classification capability was preserved as the model depth increased moderately. With a 4-layer AE, the F1-score achieved its maximum of 82%, demonstrating the best trade-off between precision and recall. Slightly lower performance was observed for 2–3 layers (81%) and 5 layers (80%), likely due to overfitting or training difficulties. These results indicate that a 4-layer configuration most effectively captures the complex patterns of the TEP while preserving generalization and robustness.

**Figure 11:**
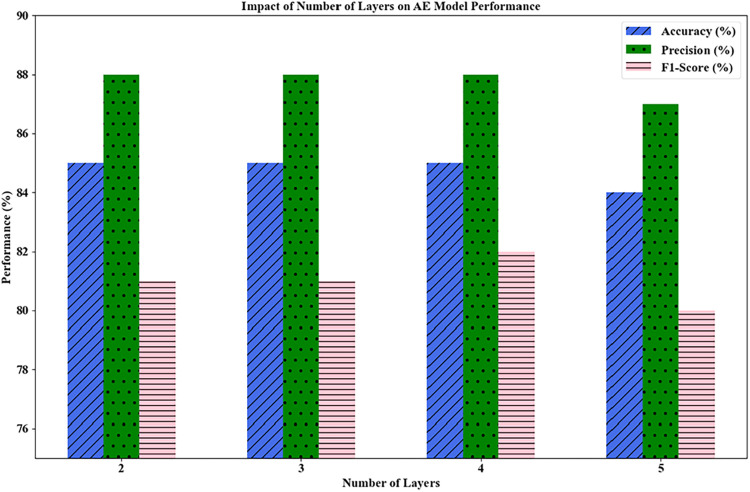
Impact of network depth on the performance of the AE model.

#### 4.6.2 The Impact of Learning Rate on Auto-Encoder Model

The learning rate is a critical hyperparameter that determines how quickly a model updates its weights during training. As shown in Figure 12, the autoencoder model exhibited improved and stable behavior as the learning rate decreased from 0.01 to 0.0001. The best performance was recorded at learning rates of 0.001 and 0.0001, where the model achieved an accuracy of 85%, precision of 88%, and F1-score of 81%. These results suggest that moderately low learning rates promote more stable convergence and better generalization, which are particularly valuable for diagnosing faults in complex and nonlinear industrial processes.

**Figure 12:**
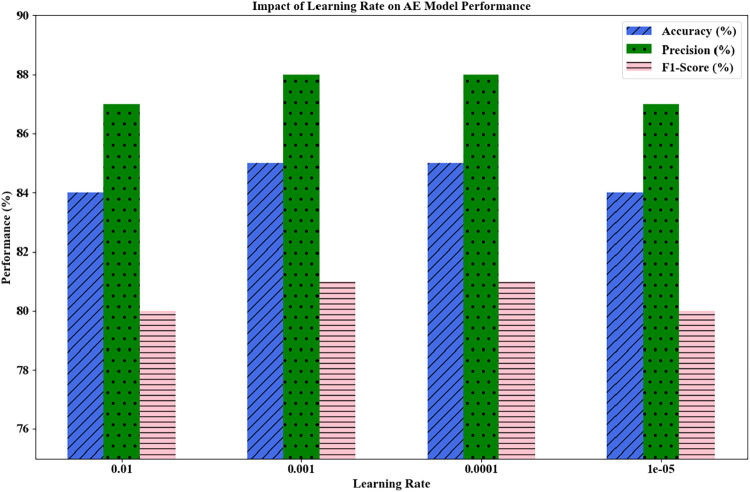
Influence of learning rate on the performance of the AE model.

However, reducing the learning rate further to 0.00001 resulted in a modest drop in accuracy (84%) and F1-score (80%), probably caused by underfitting. At such low values, the model’s weight updates become too small, slowing down the learning process and limiting its ability to capture meaningful fault-related patterns within the fixed training period. Given the high variability and noise inherent in the TEP dataset, where small anomalies in sensor readings can indicate emerging faults, learning rates of 0.001 or 0.0001 emerged as optimal. These values provided a reliable balance between training efficiency and convergence stability, enabling the AE model to effectively learn and generalize from limited and imbalanced fault data without overshooting optimal solutions. Careful learning rate selection is critical for robust fault diagnosis, as moderate values enable stable training while preserving sensitivity to subtle faults, a key requirement for safe and reliable industrial process monitoring.

#### 4.6.3 The Impact of the Number of Epochs on the Auto-Encoder Model

Similarly, the effect of training epochs on the performance of the autoencoder for fault detection in the TEP was examined. Figure 13 shows how different training durations alter critical performance measures, such as accuracy, precision, and F1-score.

**Figure 13:**
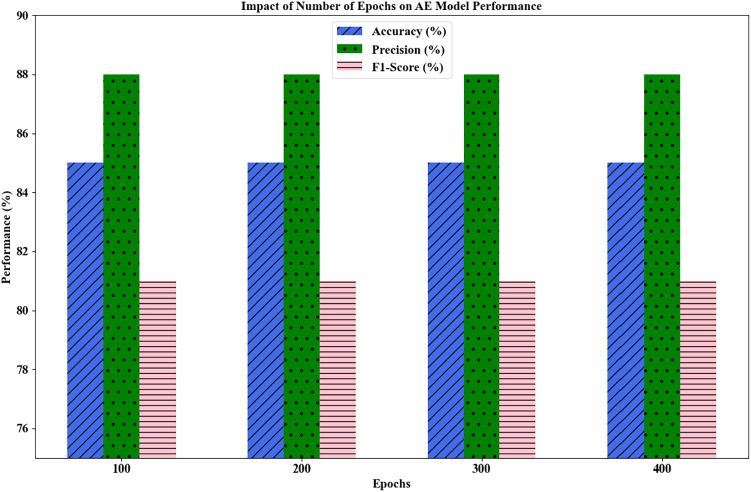
Impact of the number of epochs on the AE model performance.

The AE model maintained a consistent accuracy of 85%, precision of 88%, and F1-score of 81% across all tested epochs (100, 200, 300, and 400), revealing a stable capacity to detect and classify faults regardless of training duration. The uniformity in these metrics suggests that the model achieved convergence early during training, likely within the first 100 epochs, and that extending training beyond this point offered no additional performance benefits. This behavior indicates that while longer training durations did not harm performance, they also did not enhance the model’s ability to generalize or improve detection sensitivity. Excessive training risks overfitting, especially with noisy, imbalanced datasets like TEP. A duration of 100 epochs was sufficient for optimal AE performance, ensuring reliable detection of both common and subtle faults.

#### 4.6.4 The Impact of Dropout on Auto-Encoder Model

Dropout is commonly applied in deep learning process monitoring as a regularization strategy to prevent overfitting, particularly when fault samples are limited or classes are imbalanced. Its impact on model performance was examined under optimized training settings (Figure 14). The AE model exhibited consistent performance with dropout rates between 0.1 and 0.3 (accuracy: 84–85%; precision: 87–88%; F1-score: 80–81%), indicating that moderate dropout improves generalization while maintaining classification accuracy. When the dropout rate was raised to 0.4, the F1-score declined to 80% despite stable accuracy and precision, suggesting that too much regularization limits the model’s ability to capture key features needed for rare or subtle faults. This recall reduction is particularly significant for challenging TEP faults, including temperature step changes (Fault 3) and sticking valves (Fault 9). Dropout rates between 0.1 and 0.3 provide a better balance between robustness and sensitivity, improving fault coverage without overfitting. Systematic hyperparameter tuning is essential for AE-based FDD, with an optimal configuration of 3–4 layers, learning rates of 0.0001–0.001, 100–400 epochs, and 0.2–0.3 dropout, achieving stable and reliable performance.

**Figure 14:**
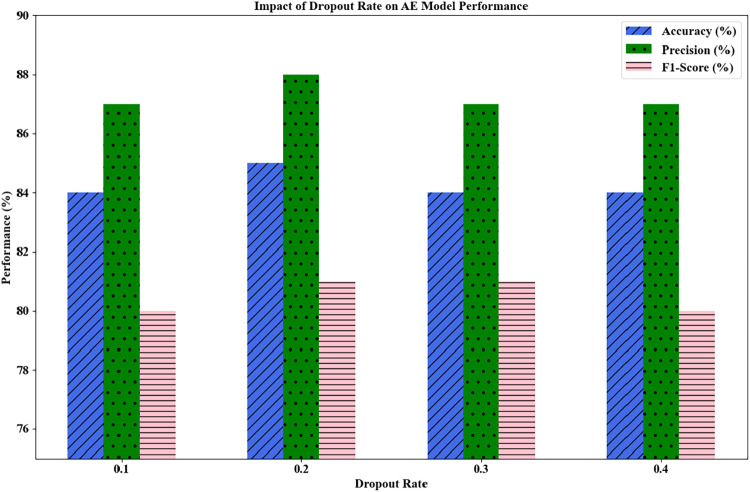
Impact of dropout rate on the AE model performance.

### 4.7 Evaluating and Comparing the Performance of the Integrated Framework

#### 4.7.1 Model Evaluation Using Confusion Matrix and ROC Analysis

The confusion matrix in Figure 15 illustrates the model’s robust diagnostic performance across all the process conditions. Pronounced diagonal dominance indicates high classification accuracy, with minimal off-diagonal dispersion reflecting low misclassification rates. The strong concentration along the main diagonal confirms the model’s ability to distinguish normal and faulty states, even under complex and overlapping sensor patterns. Minor deviations are observed for faults F9, F10, and F15, suggesting slight overlaps in their dynamic signatures. The LSTM confusion matrix is provided in Figure S1.

**Figure 15:**
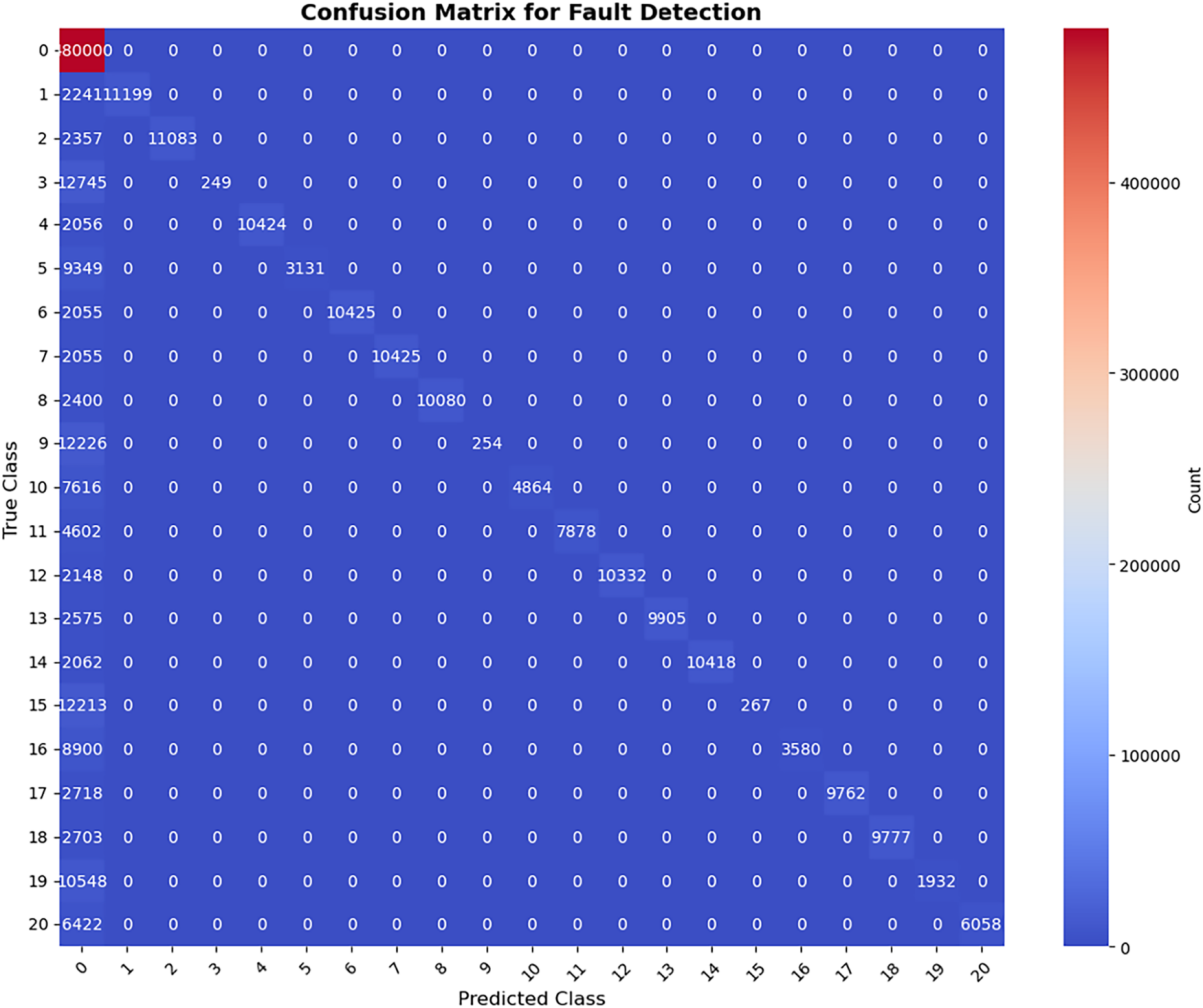
Confusion matrix showing the AE model’s classification performance.

In addition to the confusion matrix, the ROC of the DL models was analyzed to provide further insights into diagnostic performance. Figure 16 illustrates the multiclass ROC curves for fault detection across all TEP conditions. The ROC analysis indicates that most faults achieve high discriminative performance, with AUC values exceeding 0.85 for the majority of classes, demonstrating strong sensitivity and specificity. However, a few faults (e.g., Fault 3, Fault 9, Fault 15, and Fault 19) exhibit lower AUC values (0.51–0.58), likely due to overlapping dynamic patterns or weaker signal distinctiveness (Figure S2).

**Figure 16:**
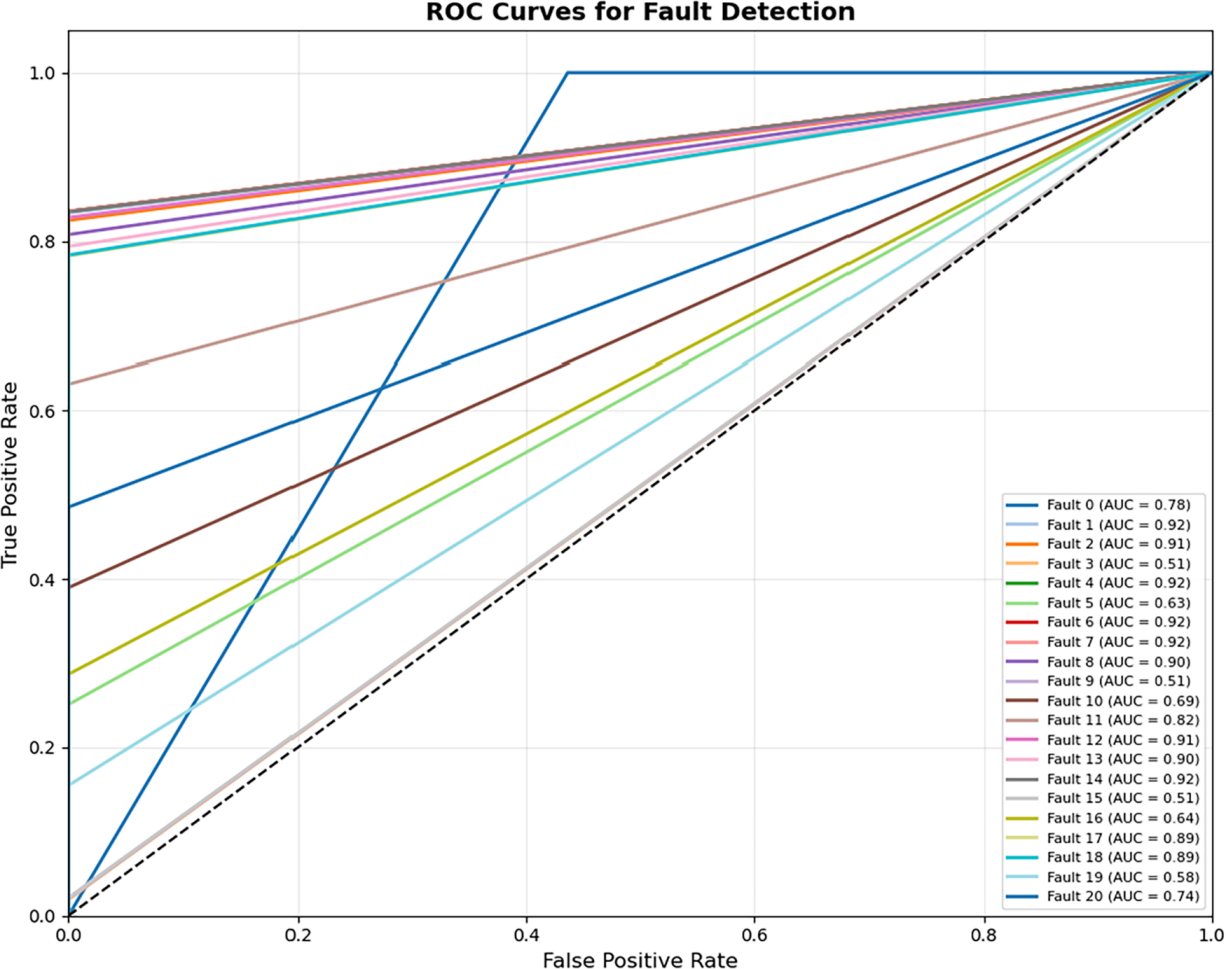
Multiclass ROC curves of the AE model for fault detection.

#### 4.7.2 Comparative Evaluation of Fault Classification Models

To further assess the fault diagnosis effectiveness of the integrated framework in complex chemical processes, comparisons were made with unsupervised machine learning methods, including PCA and OC-SVM. Table 6 presents the classification accuracies of the AE, LSTM, OC-SVM, and PCA models across 20 Tennessee Eastman process fault scenarios. The AE model achieved the highest average accuracy (85%), robustly detecting most faults, while low-variance faults (F3, F9, and F15) remained challenging. LSTM demonstrated moderate performance (70%), excelling in dynamic fault recognition but underperforming for subtle patterns. OC-SVM (58%) and PCA (44%) showed limited reliability, struggling with nonlinear faults due to sparsity and linear assumptions, respectively.

**Table 6:** Classification Accuracy for All Fault Classes

faults/models	AE	LSTM	OC-SMV	PCA
fault class	accuracy (%)	accuracy (%)	accuracy (%)	accuracy (%)
F1	83	81	84	87
F2	83	78	84	77
F3	2	50	6	1
F4	84	77	83	0
F5	25	78	30	2
F6	84	83	85	88
F7	84	83	86	84
F8	81	75	83	85
F9	2	33	9	1
F10	42	77	41	27
F11	66	70	59	22
F12	83	71	84	89
F13	80	78	81	76
F14	84	81	85	69
F15	2	12	10	0
F16	31	73	41	13
F17	79	79	71	39
F18	78	72	79	83
F19	20	78	23	5
F20	49	79	45	21
average (%)	85	70	58	44

#### 4.7.3 Fault Confidence Evaluation Using BPA and Accuracy Weighting

Table 7 presents the accuracy metrics, basic probability assignments, and weighted sum contributions (ω_1_ × ACC_1_) for the 20 TEP fault scenarios. The BPAs were derived by normalizing individual fault accuracies (∑ACC = 11.90), quantifying each fault’s relative evidence strength within the DST fusion framework, as expressed in the following equations.

**Table 7:** Accuracy Metrics, BPAs, and Weighted Sum Contributions for Each Fault Scenario

fault class	accuracy	BPAs	weighted sum
F1	0.83	*m*({*F*_1_}) = 0.0698	ω_1_ × ACC_1_ = 0.0579
F2	0.83	*m*({*F*_2_}) = 0.0698	ω_2_ × ACC_2_ = 0.0579
F3	0.02	*m*({*F*_3_}) = 0.0017	ω_3_ × ACC_3_ = 0.0000
F4	0.84	*m*({*F*4}) = 0.0706	ω4 × ACC_4_ = 0.0593
F5	0.25	*m*({*F*_5_}) = 0.0210	ω_5_ × ACC_5_ = 0.0053
F6	0.84	*m*({*F*_6_}) = 0.0706	ω_6_ × ACC_6_ = 0.0593
F7	0.84	*m*({*F*_7_}) = 0.0706	ω_7_ × ACC_7_ = 0.0593
F8	0.81	*m*({*F*_8_}) = 0.0681	ω_8_ × ACC_8_ = 0.0551
F9	0.02	*m*({*F*_9_}) = 0.0017	ω_9_ × ACC_9_ = 0.000
F10	0.42	*m*({*F*_10_}) = 0.0353	ω_10_ × ACC_10_ = 0.0148
F11	0.66	*m*({*F*_11_}) = 0.0555	ω_11_ × ACC_11_ = 0.0366
F12	0.83	*m*({*F*_12_}) = 0.0698	ω_12_ × ACC_12_ = 0.0579
F13	0.80	*m*({*F*_13_}) = 0.0672	ω_13_ × ACC_13_ = 0.0538
F14	0.84	*m*({*F*_14_}) = 0.0706	ω_14_ × ACC_14_ = 0.0593
F15	0.02	*m*({*F*_15_}) = 0.0017	ω_15_ × ACC_15_ = 0.0000
F16	0.31	*m*({*F*_16_}) = 0.0261	ω_16_ × ACC_16_ = 0.0081
F17	0.79	*m*({*F*_17_}) = 0.0664	ω_17_ × ACC_17_ = 0.0525
F18	0.78	*m*({*F*_18_}) = 0.0656	ω_18_ × ACC_18_ = 0.0511
F19	0.20	*m*({*F*_19_}) = 0.0168	ω_19_ × ACC_19_ = 0.0034
F20	0.49	*m*({*F*_20_}) = 0.0412	ω_20_ × ACC_20_ = 0.0202

Basic probability assignments for each fault class are defined as

38 $$m\left(\{F_{i}\}\right)=w_{1},w_{2},w_{3},...,n=\frac{{ACC}_{1}}{\sum {ACC}_{i}},\frac{{ACC}_{2}}{\sum {ACC}_{i}},\frac{{ACC}_{3}}{\sum {ACC}_{i}},...,n$$

The weighted sum of individual accuracies

39 $$F_{i}=\sum_{i=1}^{n}w_{i}\cdot {\mathrm{ACC}}_{i}$$

High-performing faults: F1, F2, F4, F6–F8, and F12–F14, exhibited accuracies between 0.81 and 0.84, corresponding to BPAs of 0.0681–0.0706 and weighted contributions of 0.0551–0.0593, indicating dominant evidence support and consistent diagnostic confidence. Moderate accuracies for F10, F11, F16, and F20 (0.31–0.66) yielded proportional BPAs (0.0261–0.0555) and weighted sums (0.0081–0.0366), reflecting partially separable fault characteristics. Conversely, F3, F9, and F15 demonstrated minimal accuracy (0.02) and negligible contribution (0.0000), signifying weak evidence reliability and higher uncertainty. Normalized BPA accuracy weighting quantifies fault confidence, improves interpretability, and supports prioritizing reliable diagnostics and refining weak faults. The detailed step-by-step mathematical formulation, including all intermediate values and substitutions, is provided in the Supporting Data (eqs S1–S7).

#### 4.7.4 Performance of Fault Detection Models after DST Integration

Table 8 summarizes the performance of the fault detection models before and after the application of DST–based fusion. Across all cases, DST fusion consistently enhances diagnostic performance.

**Table 8:** Evaluation of Deep Learning Fault Detection Models with and without DST Fusion

model	accuracy (%)	precision (%)	F1-score (%)
PCA	44	49	52
OC-SVM	58	50	37
LSTM	70	80	74
AE	85	88	82
PCA-DST	66	68	62
OC-SVM-DST	66	58	46
LSTM-DST	75	90	81
AE-DST	91	92	86

Notably, the AE–DST framework achieves the best results, with 91% accuracy, 92% precision, and an F1-score of 86%, demonstrating the effectiveness of DST in improving fault detection reliability. The LSTM–DST model also benefits from fusion, exhibiting increased precision (90%) and an improved F1-score (81%). Integrating deep learning with DST enhances accuracy and reliability in fault detection for complex process systems. Wang et al.^29^ reported that the outputs of four ML models were combined using DST, where the OAO-SVM achieved the highest average classification accuracy (83.2%) across ten faults in the Tennessee Eastman process, followed by SVDD (81%), BPNN (79%), and *k*-NN (73%).

To graphically illustrate the comparative performance of the models, results from four selected models are presented in Figure 17. The PCA model achieved an average classification accuracy of 44%, which increased to 66% after DST integration, representing a 22% improvement. Similarly, the AE and LSTM models exhibited accuracy gains of 6 and 5%, respectively, when fused with DST. Although formal statistical significance testing was not conducted, these improvements are consistent across the evaluated fault scenarios and are accompanied by notable increases in precision and F1-score, particularly for the AE–DST model, which showed improvements of 4% in both metrics. The observed gains are primarily attributed to the accuracy-based weighting within the DST framework, where classification accuracies are transformed into BPAs, allowing more reliable predictions to have greater influence on the final decision. The AE–DST model outperformed conventional methods by over 30% in accuracy, highlighting the robustness of deep learning–DST integration.

**Figure 17:**
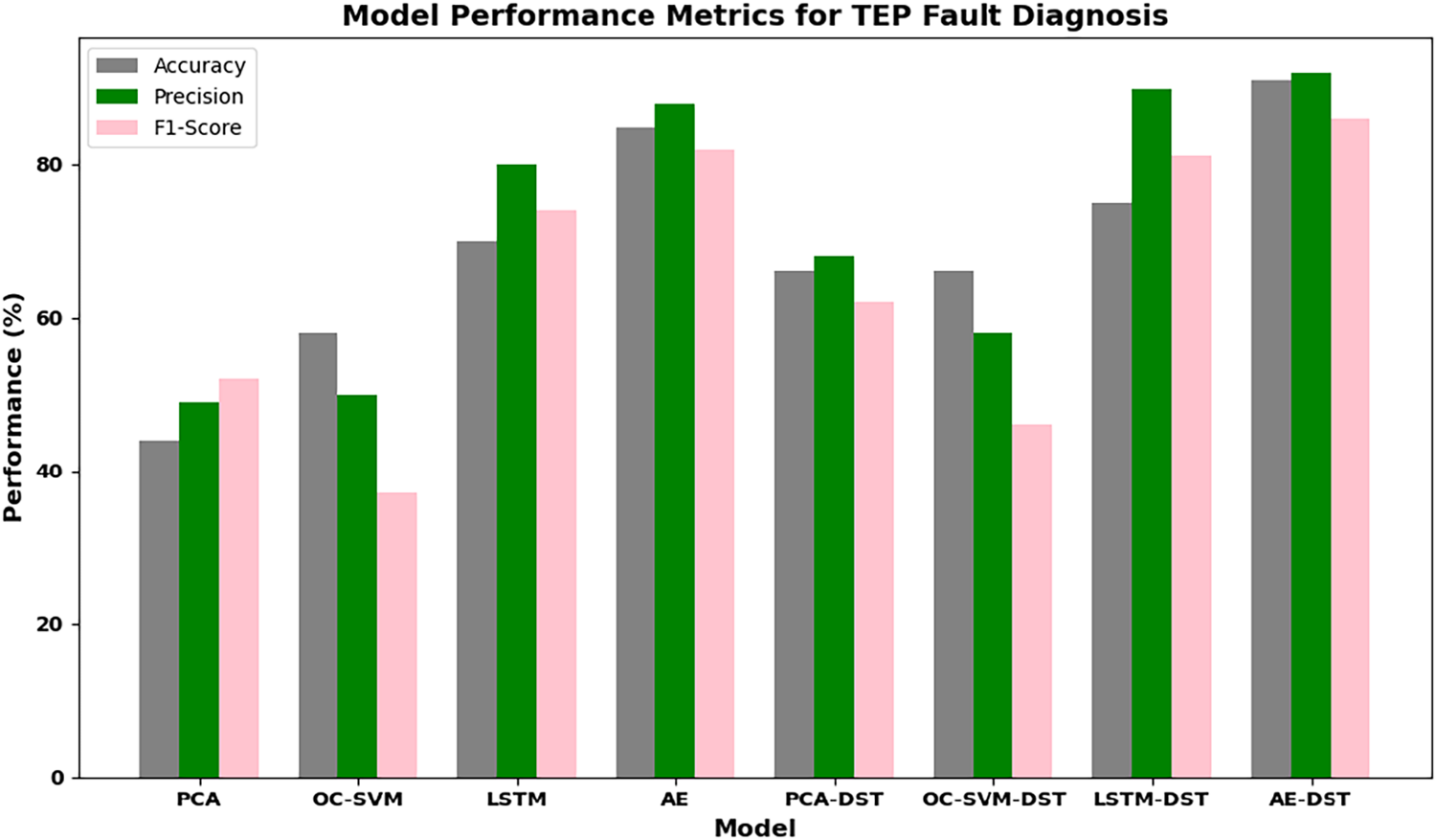
Comparison of model performance metrics for TEP fault diagnosis.

#### 4.7.5 Performance of Fault Detection Models with Attention Integration

Table 9 summarizes the results of the AE model with integrated attention mechanisms across 20 fault types in TEP. The framework achieves robust average metrics (accuracy: 90%; precision: 91%; F1 score: 90%), demonstrating effective fault detection and reliable classification. Attention integration markedly improves detection for most faults, particularly those with moderate to high prevalence (F1, F2, F4, F6, F12), where accuracy and F1 scores exceed 80% (Table S7).

**Table 9:** Evaluation of the AE Fault Detection Model with Attention Integration

fault class	accuracy (%)	precision (%)	F1-score (%)
F1	83	95	91
F2	84	98	90
F3	2	5	5
F4	84	96	91
F5	27	45	41
F6	86	96	93
F7	84	96	91
F8	81	97	89
F9	2	5	5
F10	38	60	55
F11	62	84	77
F12	83	98	92
F13	79	95	90
F14	84	95	91
F15	3	5	5
F16	28	48	43
F17	78	91	88
F18	79	90	89
F19	14	26	24
F20	49	72	67
average (%)	90	91	90

#### 4.7.6 Interpretability and Model Transparency

The feature attention heatmap reveals that certain variables, particularly XMEAS(12) (separator level), XMEAS(40) (product component G), and XMEAS(41) (product component H), consistently receive higher attention across multiple faults, indicating their critical role in capturing process deviations (Figure 18). Faults with strong detection metrics, including F1 (A/D feed ratio changes, step), F2 (B feed ratio changes, step), F6 (A feed loss, step), and F12 (slow drift in the inlet temperature of the separator’s cooling water), exhibit concentrated attention on specific subsets of variables, while more challenging faults, such as F3 (D feed temperature changes, step), F9 (D feed temperature changes, random variation), and F15 (separator valve sticking), show uniformly low attention. This demonstrates that the AE model selectively emphasizes fault-relevant features, improving detection accuracy and interpretability.

**Figure 18:**
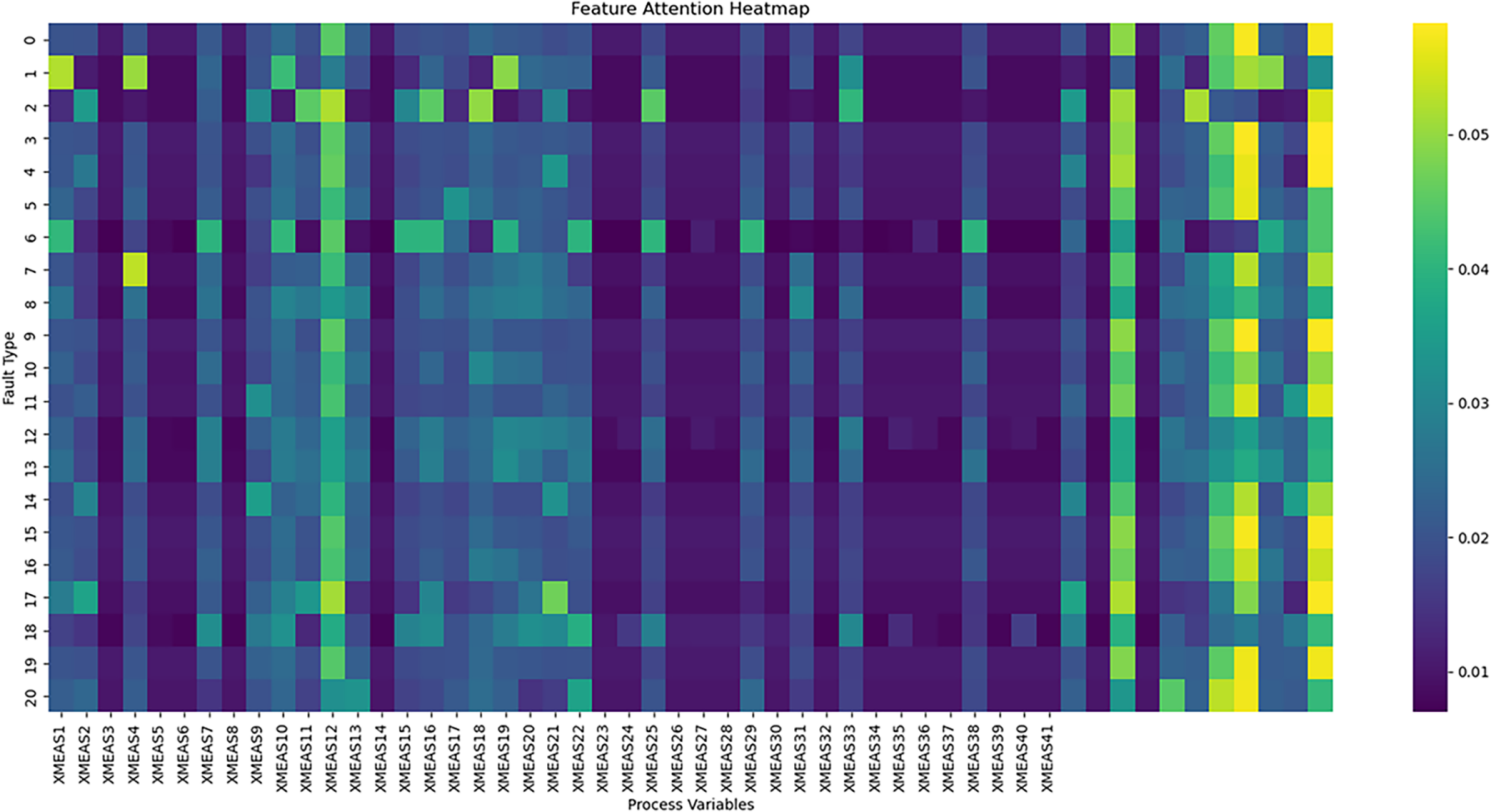
Feature attention heatmap of the AE model for Tennessee Eastman process variables.

The fault-wise attention analysis (Table 10) reveals that XMEAS2 (D feed stream 2) and XMEAS25 (reactor feed component C) consistently receive the highest attention across all 20 faults, with XMEAS19 (stripper steam flow) and XMEAS11 (separator temperature) moderate and XMEAS8 (reactor level) and XMEAS13 (separator pressure) lower but steady, indicating primary and secondary roles in fault detection. These quantitative results complement Figure 18, which visually depicts global attention trends across all of the 41 TEP variables. While the heatmap shows overall attention patterns and feature suppression, Table 10 isolates and numerically validates the contributions of the most influential features. Together, they provide a comprehensive interpretability framework combining global pattern analysis with faultwise quantification.

**Table 10:** Fault-Wise Attention Weights of Dominant Variables in the Tennessee Eastman Process

fault class	XMEAS2	XMEAS25	XMEAS19	XMEAS11	XMEAS8	XMEAS13
F1	0.352	0.209	0.172	0.165	0.045	0.034
F2	0.357	0.205	0.173	0.165	0.045	0.034
F3	0.357	0.208	0.172	0.165	0.043	0.034
F4	0.354	0.209	0.170	0.165	0.044	0.035
F5	0.352	0.205	0.173	0.166	0.045	0.036
F6	0.353	0.209	0.170	0.165	0.045	0.036
F7	0.355	0.206	0.172	0.165	0.045	0.035
F8	0.354	0.207	0.174	0.165	0.045	0.035
F9	0.353	0.208	0.171	0.164	0.045	0.036
F10	0.354	0.210	0.170	0.165	0.045	0.035
F11	0.353	0.206	0.172	0.166	0.045	0.036
F12	0.356	0.207	0.170	0.164	0.045	0.036
F13	0.356	0.209	0.169	0.165	0.044	0.035
F14	0.353	0.206	0.173	0.166	0.045	0.035
F15	0.355	0.209	0.173	0.164	0.044	0.033
F16	0.353	0.206	0.73	0.166	0.045	0.035
F17	0.354	0.206	0.173	0.166	0.044	0.035
F18	0.356	0.206	0.172	0.165	0.045	0.035
F19	0.357	0.207	0.171	0.165	0.044	0.034
F20	0.356	0.205	0.173	0.166	0.044	0.034

## 5 Conclusion and Future Research Directions

A robust and interpretable framework for fault diagnosis in complex chemical processes is proposed, integrating deep learning models (AE and LSTM) with the DST and feature attention mechanisms. By harnessing the representation learning capability of deep learning, the uncertainty management strength of DST, and the interpretability provided by feature attention, the framework effectively addresses key challenges in process monitoring, including sensor noise, conflicting diagnostic evidence, fault uncertainty, and limited transparency in decision-making. Systematic tuning of critical hyperparameters, namely, the number of layers, learning rate, training epochs, and dropout rate, enabled the models to achieve robust and stable performance across diverse fault conditions.

Experimental validation using the Tennessee Eastman process demonstrates that the proposed framework outperforms standard models. AE–DST model achieved the best overall performance, with 91% accuracy, 92% precision, and an F1-score of 86%, while the LSTM–DST model also showed consistent performance improvements. Furthermore, the attention-enhanced AE fault detection model achieved strong average metrics across 20 fault classes, attaining 90% accuracy, 91% precision, and 90% F1-score. Importantly, the interpretability provided by the feature attention mechanism differs from hierarchical frameworks reported in ref 30. While hierarchical methods identify fault locations at the process-section level, the proposed framework provides variable-level interpretability by highlighting key process variables and temporal features driving diagnostic decisions. This offers more detailed and complementary insights, enhancing diagnostic transparency and practical usability. Furthermore, attention heatmaps and fault-wise analysis reveal that specific process variables consistently receive higher attention, confirming their critical role in fault detection and supporting informed sensor prioritization.

Comparative evaluations against traditional unsupervised methods, including PCA and one-class SVM (OC-SVM), both with and without DST, further confirm the advantage of deep-learning-based models in capturing nonlinear fault patterns and reducing diagnostic uncertainty. This framework delivers a robust and scalable solution for the real-time monitoring of chemical processes, enhancing safety, reliability, and overall operational efficiency. Future research will focus on extending the proposed framework to other industrial benchmark processes, such as continuous stirred tank reactors, crude distillation columns, and fed-batch fermentation systems, to further evaluate its generalizability and adaptability under various operational conditions. Additional research directions include integrating adaptive attention mechanisms and developing strategies for real-time industrial deployments.

## Supplementary Material



## Notes

Definitions of Dempster–Shafer theory notations; list of measured process variables (TEP dataset); autoencoder hyperparameter sensitivity analysis (number of layers, learning rate, epochs, dropout); LSTM model performance across 20 fault classes; confusion matrix and multiclass ROC curves; and detailed step-by-step derivation of basic probability assignment (PDF)

## †

Department of Chemical Engineering, Faculty of Industrial Technology and System Engineering, Institut Teknologi Sepuluh Nopember (ITS), 60111 Surabaya, Indonesia

## Abbreviations

AI: artificial intelligence
AE: autoencoder
DL: deep learning
GPU: graphics processing unit
ML: machine learning
PCA: principal component analysis
RNN: recurrent neural network
Adam: adaptive moment estimation
DNN: deep neural network
DST: Dempster–Shafer theory
DFN: deep feedforward network
SDAE: stacked denoising autoencoder
ANN: artificial neural network
KPLS: kernel partial least-squares
OC-SVM: one-class support vector machine
KPCA: kernel principal component analysis
FOD: frame of discernment
LSTM: long short-term memory
SGD: stochastic gradient descent
MSE: mean squared error
MLP: multilayer perceptron
ReLU: rectified linear unit
BPA: basic probability assignments
SVM: support vector machine
DL-DST: deep learning Dempster–Shafer theory
TEP: Tennessee Eastman process
XMV: manipulated variable
PLS: partial least-squares
XMEAS: measured variable
FDA: Fisher discriminant analysis
CCA: canonical correlation analysis
DBN: deep belief network
SSAE: stacked sparse autoencoders
CNN: convolutional neural network
SSAE: stacked supervised autoencoders
AHP: analytic hierarchy process
ER: evidential reasoning
DCRNN: deep convolutional recurrent neural network
FDR: fault detection rate
kNN: *k*-nearest neighbor
LSS: LSTM-based statistical surveillance
PDNN: process dynamics-guided neural network
GGMAE: graph-guided masked autoencoder
BPNN: back-propagation artificial neural network
SVDD: support vector data description
OAO-SVM: one-against-one SVM
*k*-NN: *k*-nearest neighbors
