# Supplementary material for: Robust and Interpretable Deep Learning Fault Diagnosis in Complex Chemical Processes: Performance Enhancement via Dempster–Shafer Theory and Feature Attention
Source: ACS Omega. 2026 Jul 9;11(28):41772–93. doi: 10.1021/acsomega.6c01554 (PMC13393364; doi:10.1021/acsomega.6c01554)
Supplement: Supplementary file 1 [file ao6c01554_si_001.pdf]

Supplementary Material

Robust and Interpretable Deep Learning Fault Diagnosis in Complex Chemical Processes: Performance Enhancement Via Dempster–Shafer Theory and Feature Attention

Remigius Nnadozie Ewuzie [a], Shivaneswar Gunasekaran [a], Zainal Ahmad [a],

Juwari Juwari [a, †,] Norazwan Md Nor [a,*]

[a] School of Chemical Engineering, Engineering Campus, Universiti Sains Malaysia, 14300 Nibong Tebal, Penang, MALAYSIA.

[†] Current address: Department of Chemical Engineering, Faculty of Industrial Technology and System Engineering, Institut Teknologi Sepuluh Nopember (ITS), 60111 Surabaya, Indonesia.

*Email: chnorazwan@usm.my (corresponding author)

Tel: +604-5995750

Table S1. **Definitions of Key Notations Employed in Dempster–Shafer Theory**

Notation	Term	Definition
Θ	frame of discernment	Complete set of mutually exclusive hypotheses representing system states (e.g., normal and faulty conditions).
$F \subseteq \Theta$	Hypothesis (focal element)	A subset of possible faults.
$m(A)$	Basic probability assignment (BPA) (or mass function)	The belief mass assigned to hypothesis A.
$m(\{F_i\})$	Singleton BPA	Belief mass assigned only to a single fault F_i.
$\sum_{A \subseteq \Theta} m(A) = 1$	Normalization rule	The total mass over all subsets of Θ equals 1.
$m(\emptyset) = 0$	Empty set constraint	No mass is assigned to the empty set.
K	Conflict coefficient	Total conflicting mass between evidence sources
$Bel(A)$	Belief function	Measures total belief that strictly supports subset A of Θ.
$Pl(A)$	Plausibility function	Reflects the maximum belief that could support A.
m_1, m_2	Independent mass functions	Represent independent evidence sources defined over Θ

Table S2. The Measured Process Variables

Process variables	Process description	Process variables	Process description
XMEAS(1)	A feed stream 1	XMEAS(22)	Separator cooling water outlet temperature
XMEAS(2)	D feed stream 2	XMEAS(23)	Reactor feed component A
XMEAS(3)	E feed stream 3	XMEAS(24)	Reactor feed component B
XMEAS(4)	Total feed stream 4	XMEAS(25)	Reactor feed component C
XMEAS(5)	Recycle flow	XMEAS(26)	Reactor feed component D
XMEAS(6)	Reactor feed rate	XMEAS(27)	Reactor feed component E
XMEAS(7)	Reactor pressure	XMEAS(28)	Reactor feed component F
XMEAS(8)	Reactor level	XMEAS(29)	Purge component A
XMEAS(9)	Reactor temperature	XMEAS(30)	Purge component B
XMEAS(10)	Purge rate	XMEAS(31)	Purge component C
XMEAS(11)	Separator temperature	XMEAS(32)	Purge component D
XMEAS(12)	Separator level	XMEAS(33)	Purge component E
XMEAS(13)	Separator pressure	XMEAS(34)	Purge component F
XMEAS(14)	Separator underflow	XMEAS(35)	Purge component G
XMEAS(15)	Stripper level	XMEAS(36)	Purge component H
XMEAS(16)	Stripper pressure	XMEAS(37)	Product component D
XMEAS(17)	Stripper underflow	XMEAS(38)	Product component E
XMEAS(18)	Stripper temperature	XMEAS(39)	Product component F
XMEAS(19)	Stripper steam flow	XMEAS(40)	Product component G
XMEAS(20)	Compressor work	XMEAS(41)	Product component H
XMEAS(21)	Reactor cooling water outlet temperature		

Table S3. **The Impact of the Number of Layers on the Autoencoder Model**

Number of layers	Accuracy (%)	Precision (%)	F1-Score (%)
2	85	88	81
3	85	88	81
4	85	88	82
5	84	87	80

Table S4. The Impact of Learning Rate on the Autoencoder Model

Learning rate	Accuracy (%)	Precision (%)	F1-Score (%)
0.01	84	87	80
0.001	85	88	81
0.0001	85	88	81
0.00001	84	87	80

Table S5. The Impact of the Number of Epochs on the Autoencoder Model

Epochs	Accuracy (%)	Precision (%)	F1-Score (%)
100	85	88	81
200	85	88	81
300	85	88	81
400	85	88	81

Table S6. **The Impact of Dropout on the Autoencoder Mode**

Dropout	Accuracy (%)	Precision (%)	F1-Score (%)
0.1	84	87	80
0.2	85	88	81
0.3	84	87	81
0.4	84	87	80

Table S7. Evaluation of the LSTM Fault Detection Model with Attention Integration

Fault class	Accuracy (%)	Precision (%)	F1-Score (%)
F1	82	97	89
F2	81	99	89
F3	17	7	10
F4	24	17	20
F5	13	26	17
F6	83	92	87
F7	81	91	86
F8	59	75	66
F9	23	13	16
F10	51	34	41
F11	31	34	32
F12	62	67	65
F13	64	85	73
F14	75	88	81
F15	1	7	2
F16	22	13	16
F17	64	55	59
F18	64	92	76
F19	46	33	38
F20	36	36	36
Average (%)	79	84	79

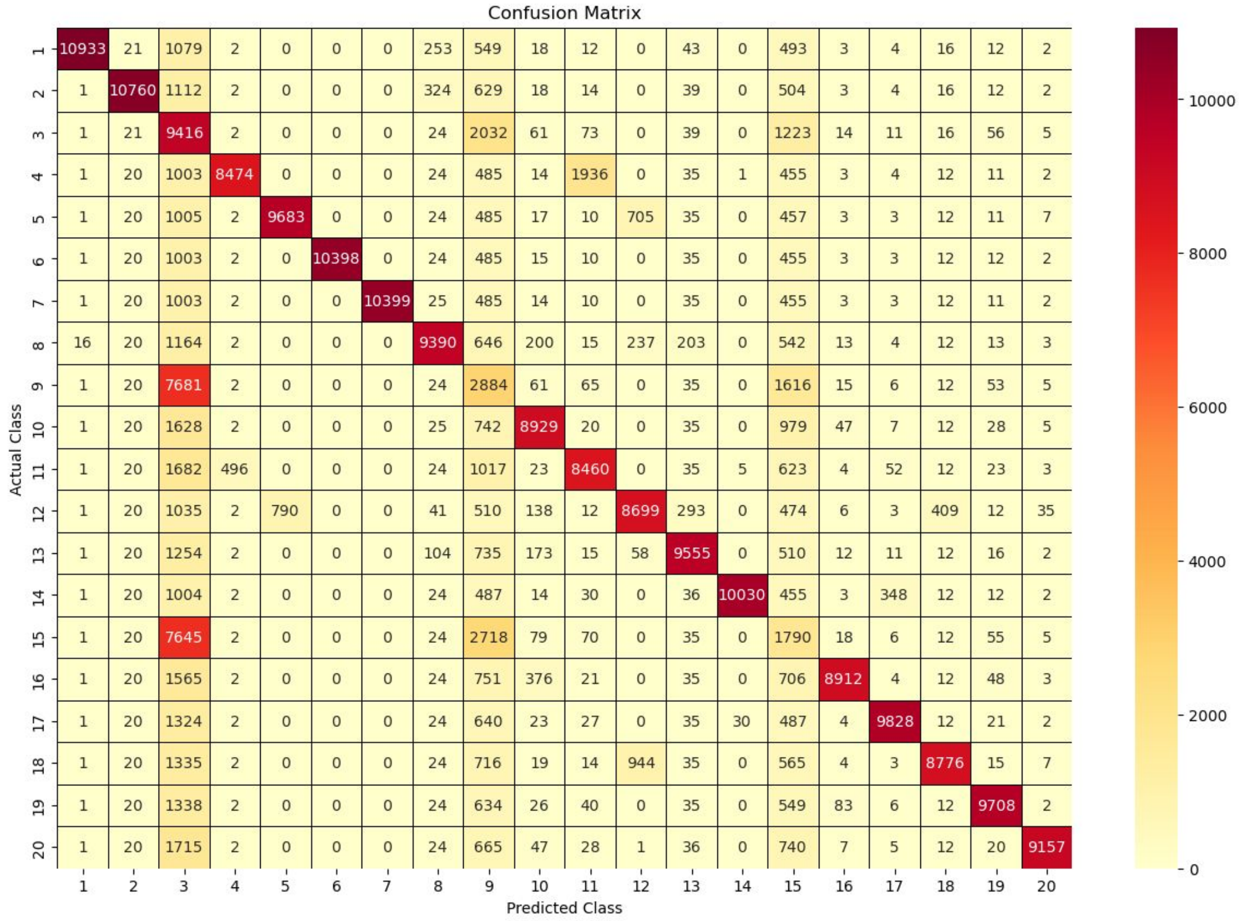

Figure S1. Confusion matrix of the LSTM-based fault classification model.

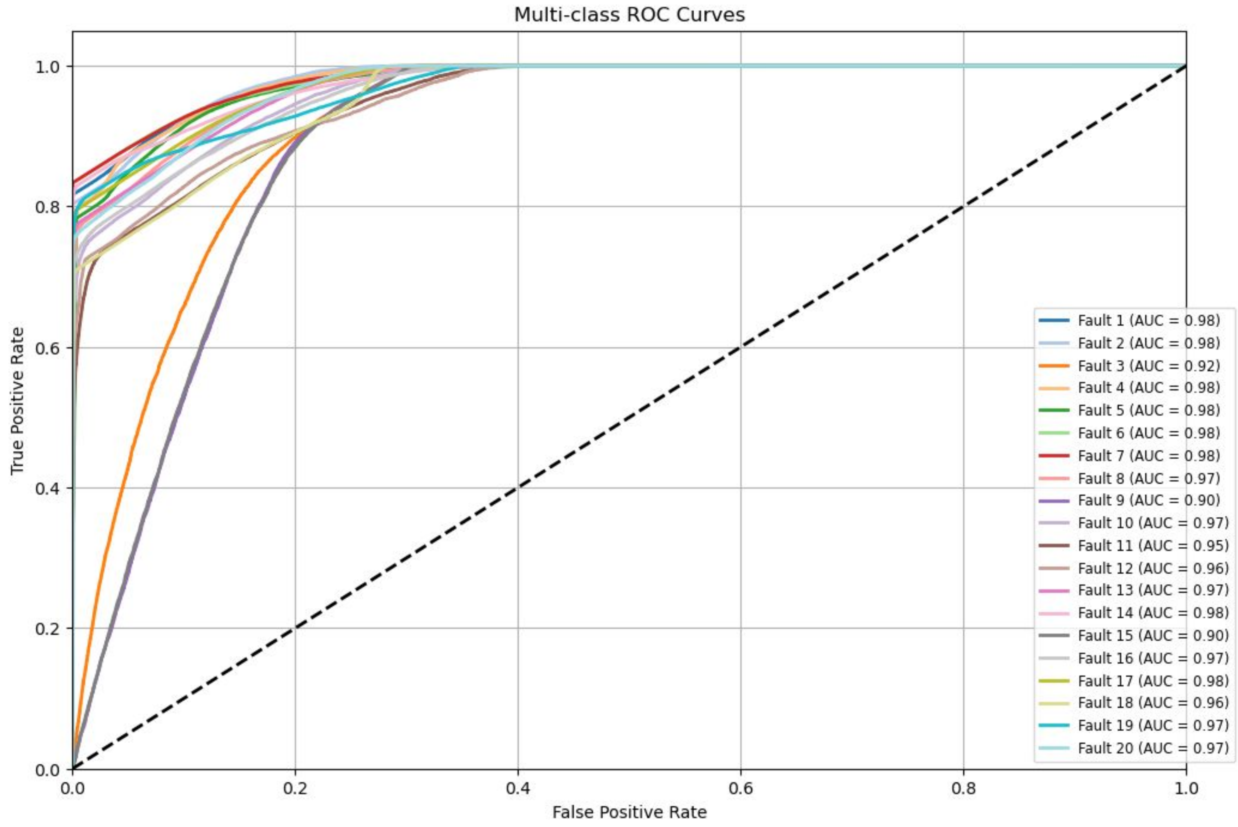

Figure S2. Multi-class ROC curves of the LSTM model for fault detection.

Mathematical formulation

The BPA formulation presented in **Eqs** (**38**) and (**39**) of the main manuscript is expanded here step by step.

ACC_i represents the classification accuracy of the fault class F_i where $i = 1, 2,3,\ldots.n$ and n the total number of fault classes (20).

Classification accuracy of fault class F_i:

$$ACC_1,\ ACC_2,\ ACC_3,\ldots\ldots..20 = 83\%, 83\%, 2\%,\ldots.49\% \qquad S1$$

Convert percentages into decimal values:

$$ACC_1,\ ACC_2,\ ACC_3,\ldots\ldots..20 = 0.83, 0.83, 0.02,\ldots.0.49 \qquad S2$$

The sum of the total classification accuracy:

$$\sum_{i=1}^{n} Acc_i = 0.83 + 0.83 + 0.02 + \ \ldots.. + 0.49 = 11.90 \qquad S3$$

Basic probability assignments for each fault class are defined as:

$$m(\{F_i\}) = w_{1,}\ w_2\ w_3,\ldots.n = \frac{ACC_1}{\sum ACC_i}, \frac{ACC_2}{\sum ACC_i}, \frac{ACC_3}{\sum ACC_i},\ldots.n \qquad S4$$

$$m(\{F_i\}) = \frac{0.83}{11.90}, \frac{0.83}{11.90}, \frac{0.02}{11.90}, \ldots \frac{0.49}{11.90} \qquad S5$$

$$m(\{F_i\}) = 0.0698, 0.0698, 0.0017,\ldots.0.0412 \qquad S6$$

where, $m(\{F_i\})$ is combined belief mass for fault class F_i *and* w_i is weight of each fault

The weighted sum of individual accuracies:

$$F_i = \sum_{i=1}^{n} w_i . ACC_i \qquad S7$$
